# Large-scale cranial window for in vivo mouse brain imaging utilizing fluoropolymer nanosheet and light-curable resin

**DOI:** 10.1038/s42003-024-05865-8

**Published:** 2024-03-04

**Authors:** Taiga Takahashi, Hong Zhang, Masakazu Agetsuma, Junichi Nabekura, Kohei Otomo, Yosuke Okamura, Tomomi Nemoto

**Affiliations:** 1 Division of Biophotonics, National Institute for Physiological Sciences, National Institutes of Natural Sciences, Higashiyama 5-1, Myodaiji, Okazaki, Aichi 444-8787 Japan; 2 Biophotonics Research Group, Exploratory Research Center on Life and Living Systems (ExCELLS), National Institutes of Natural Sciences, Higashiyama 5-1, Myodaiji, Okazaki, Aichi 444-8787 Japan; 3 School of Life Science, The Graduate University for Advanced Studies (SOKENDAI), Higashiyama 5-1, Myodaiji, Okazaki, Aichi 444-8787 Japan; 4 Department of Medical and Robotic Engineering Design, Faculty of Advanced Engineering, Tokyo University of Science, 6-3-1 Niijuku, Katsushika, Tokyo 125-8585 Japan; 5 Micro/Nano Technology Center, Tokai University, 4-1-1 Kitakaname, Hiratsuka, Kanagawa 259-1292 Japan; 6 School of Chemical Engineering and Technology, Tianjin University, 135 Yaguan Road, Jinnan District, Tianjin, 300350 China; 7 Division of Homeostatic Development, National Institute for Physiological Sciences, National Institutes of Natural Sciences, Okazaki, 444-8585 Japan; 8 Quantum Regenerative and Biomedical Engineering Team, Institute for Quantum Life Science, National Institutes for Quantum Science and Technology (QST), Anagawa 4-9-1, Chiba Inage-ku, Chiba, 263-8555 Japan; 9 Department of Biochemistry and Systems Biomedicine, Graduate School of Medicine, Juntendo University, 2-1-1 Hongo, Bunkyo-ku, Tokyo 113-8421 Japan; 10 Department of Applied Chemistry, School of Engineering, Tokai University, 4-1-1 Kitakaname, Hiratsuka, Kanagawa 259-1292 Japan; 11 Course of Applied Science, Graduate School of Engineering, Tokai University, 4-1-1 Kitakaname, Hiratsuka, Kanagawa 259-1292 Japan

**Keywords:** Nanofabrication and nanopatterning, Cellular neuroscience, Multiphoton microscopy, Fluorescence imaging, Ca2+ imaging

## Abstract

Two-photon microscopy enables in vivo imaging of neuronal activity in mammalian brains at high resolution. However, two-photon imaging tools for stable, long-term, and simultaneous study of multiple brain regions in same mice are lacking. Here, we propose a method to create large cranial windows covering such as the whole parietal cortex and cerebellum in mice using fluoropolymer nanosheets covered with light-curable resin (termed the ‘Nanosheet Incorporated into light-curable REsin’ or NIRE method). NIRE method can produce cranial windows conforming the curved cortical and cerebellar surfaces, without motion artifacts in awake mice, and maintain transparency for >5 months. In addition, we demonstrate that NIRE method can be used for in vivo two-photon imaging of neuronal ensembles, individual neurons and subcellular structures such as dendritic spines. The NIRE method can facilitate in vivo large-scale analysis of heretofore inaccessible neural processes, such as the neuroplastic changes associated with maturation, learning and neural pathogenesis.

## Abstract (web summary)

The authors propose a new method for two-photon microscopy that can produce large cranial windows covering both the cerebrum and cerebellum of mice. The windows maintain transparency for over 5 months and allow multi-scale two-photon imaging of neuronal structures and functions in awake mice.

## Introduction

Coordinated neural activity across multiple brain regions, such as the cerebral cortex and the cerebellum, underlies higher-order brain functions^1–5^. Such activity can be measured using a variety of neuroimaging modalities, such as functional magnetic resonance imaging (fMRI), and these modalities have even been adapted to the study of various small animal models. However, while such methods enable the imaging of brain activity over broad regions, temporal and spatial resolution are limited. Alternatively, multi-electrode arrays such as Neuropixels probes provide electrophysiological recordings from many neurons with high temporal and single-cell resolution^6–8^. However, these methods inevitably are invasive due to the surgical implantation of electrodes into the brain. Furthermore, monitoring multiple brain regions using these probes increases the damage to living tissue.

Two-photon microscopy has been used to visualize neural structures and activities in various brain regions of living mammals at cellular and subcellular resolution with low invasiveness^9–13^. Recently, a variety of techniques have been described for in vivo two-photon imaging of multiple brain regions utilizing a high-power laser^14–16^, an objective lens with low magnification and a high numerical aperture (NA)^17–20^, optic devices^21–24^, multiplexing of laser pathways^17,20,25–28^. To perform high-resolution imaging in a living mouse brain, maximizing the capabilities of two-photon microscopy, a cranial window is typically created by removing a portion of the skull (craniectomy) and sealing the hole with a glass coverslip^29,30^. However, conventional cranial windows are usually small (2–4 mm in diameter) to minimize bleeding, avoid damage to large blood vessels, and prevent mechanical stress on curved brain tissue caused by a flat glass coverslip^31^. To overcome the limitations of conventional cranial windows, novel methods have been proposed such as the utilization of curved glass or polymers that can conform to the shape of the living brain surface^31–33^. However, methods have yet to be developed for the creation of larger cranial windows sufficient to simultaneously image distant regions of the cerebral cortex or cerebral cortex and cerebellum. For instance, it has proven difficult to make large cranial windows in regions near thick blood vessels, such as the sagittal sinus and transverse sinus, due to bleeding during or following craniectomy.

Previously, we proposed the use of a polyethylene-oxide-coated CYTOP (PEO-CYTOP) nanosheet as a sealing material for the creation of a large cranial window^34^. These nanosheets possess unique properties amenable to in vivo two-photon microscopy, including high adhesion strength, flexibility, and transparency. Furthermore, nanosheets with ≤200-nm thick exhibit strong physical absorption on the object surface, making them useful for surgical application^35–39^ and bioimaging^34,40–43^. To apply a PEO-CYTOP nanosheet to a living mouse brain, only the outermost surface of the CYTOP nanosheet, which is direct contact to the brain surface, was hydrophilized using polyethylene-oxide (PEO) to reduce inflammatory reactions. These PEO-CYTOP nanosheets permit the construction of cranial windows covering a substantial proportion of the parietal region, and enabled in vivo two-photon imaging of neural structures with a broad field of view (FOV) at high spatial resolution. However, these flexible PEO-CYTOP nanosheets did not suppress motion artifacts from respiratory and body movements in awake conditions. Moreover, PEO-CYTOP nanosheets could not be used for long-term imaging, such as over a few weeks of observations, due to changes in intracranial pressure and progressive damage during mouse behaviors.

In this study, we proposed a new method utilizing a biocompatible nanosheet incorporated into light-curable resin (NIRE method) to construct large cranial windows over the cortex and cerebellum suitable for long-term imaging in awake mice. The light-curable resin was applied to fix the bioinert PEO-CYTOP nanosheet onto the brain surface, regardless of surface shape and curvature. The NIRE method produced windows that maintained transparency for >5 months and suppressed motion artifacts. In addition, the NIRE method successfully enabled the visualization of neural structures and intracellular Ca^2+^ concentration changes at various scales, from populations of over a thousand neurons to single spines, in living mouse brain.

## Results

### Creation of large cranial windows using PEO-CYTOP nanosheets and light-curable resin (the NIRE method)

**Fig. 1:**
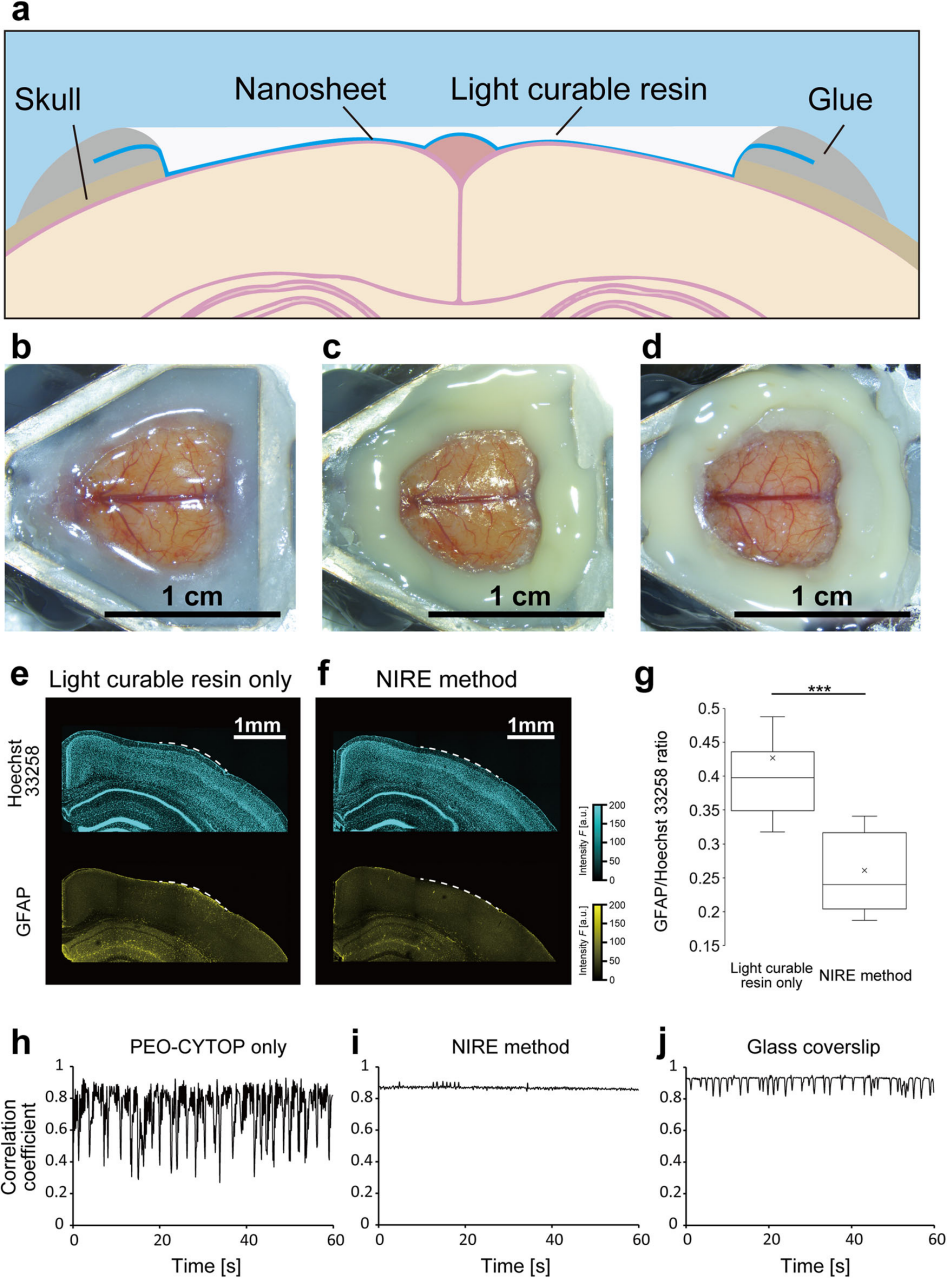
Construction of cranial windows from PEO-CYTOP nanosheets and light-curable resin. **a** Fixing the light curable resin on the PEO-CYTOP nanosheet. Schematic illustration of the ‘nanosheet improved by light-curable resin (NIRE)’ method. **b** Exposure of the dura surface by craniectomy. **c** Sealing the brain surface with a PEO-CYTOP nanosheet. **d** Fixing light-curable resin on the PEO-CYTOP nanosheet. **e** Immunostaining of astrocytes using anti-GFAP and nuclear counterstaining in a brain slice obtained 4 weeks after craniectomy and covering with light-curable resin only (without a PEO-CYTOP nanosheet). The white dashed line indicates the region where the skull was removed, and the resin was fixed. **f** Immunostaining of astrocytes using anti-GFAP and nuclear counterstaining in a brain slice obtained 4 weeks after craniectomy and the NIRE method. The white dashed line indicates the region where the skull was removed, and the resin was fixed. **g** The ratio of mean GFAP to Hoechst 33258 signal per 300 × 300 µm^2^ (15 ROIs from three mice in each condition). ***p < 0.005 by Welch’s *t* test. **h** Time series of a correlation coefficient calculated from two-photon images of SR101-labeled astrocytes acquired through a cranial window consisting of a PEO-CYTOP nanosheet in the awake condition. **i** Time series of a correlation coefficient calculated from two-photon images of SR101-labeled astrocytes acquired through a cranial window produced using the NIRE method in the awake condition. **j** Time series of a correlation coefficient calculated from two-photon images of SR101-labeled astrocytes acquired through a cranial window using the glass coverslip method in the awake condition.

The NIRE method was devised to create large cranial windows suitable for long-term two-photon imaging of widely distributed neurons in living mouse brain, including in the awake state, while suppressing motion artifacts compared to the PEO-CYTOP nanosheet only (Fig. 1a). The mouse brain surface was first exposed (Fig. 1b) and covered with a PEO-CYTOP nanosheet with a thickness of ~130 nm (Fig. 1c). Next, the surface of the PEO-CYTOP nanosheet was coated with light-curable resin and irradiated with ultraviolet (UV) light (Fig. 1d), yielding a large cranial window that strongly adhered to the brain surface and conformed to its complex surface structure.

One potential problem of UV light curing is heating damage, so the fixing procedure was first optimized using a fast-curing resin “NOA83H” and a programmable UV irradiator composed of a UV-LED and LED control board (Supplementary Fig. 1a). To evaluate temperature change induced in the light-curable resin by curing protocol, we inserted the digital thermometer electrodes (TR-71nw, T&D Corporation., Japan) into the center of the mass of light-curable resin placed in a 35 mm petri dish, just under the irradiation laser. Using this set-up, we recorded temperature changes before, during, and after UV irradiation for two irradiating patterns (Supplementary Fig. 1b, c). We found that, intermittent irradiation enhanced the temperature to below 35 °C, indicating that the light-curable resin could be cured at a temperature that was not harmful to living tissues. In contrast, a steep temperature rise occurred under continuous irradiation. Thus, we adopted intermittent high-intensity irradiation to cure the light-curable resin in the NIRE method.

Additionally, it is also possible that these window components induce a neuroinflammatory response. For evaluation of NIRE method-induced neuroinflammation, activated astrocytes were immunostained for GFAP in brain sections from mice receiving light-curable resin alone after craniectomy (Fig. 1e) or the complete NIRE method (Fig. 1f). The number of activated astrocytes was significantly lower following the NIRE method compared to application of light-curable resin alone (Fig. 1g). The NIRE method group did not show significant inflammation in the ipsilateral and the contralateral brain regions. However, these regions in the light-curable resin group (without the PEO-CYTOP nanosheets) showed significant inflammation (Supplementary Fig. 2). These results suggest that the PEO-CYTOP nanosheet acted as a barrier film to protect against inflammation induced by light-curable resin.

Next, we verified the optical properties of cranial windows produced using the NIRE method. The transmittance of the PEO-CYTOP nanosheet and the light-curable resin was approximately 100% in the wavelength range of 400–1000 nm, indicating its optical transparency and suitability for in vivo imaging (Supplementary Fig. 3). To evaluate the point-spread function (PSF) in the NIRE method, we obtained fluorescent images of 200-nm fluorescent yellow–green beads embedded at depths of 200, 500, and 1000 µm within agarose gels covered with or without the film made of the light-curable resin with a thickness of ~150 μm (Supplementary Figs. 4 and 5). The lateral full width at half maximum (FWHM) of the PSF through the light-curable resin was 544 ± 6 nm at 200 µm, 559 ± 3 nm at 500 µm, and 616 ± 6 nm at 1000 µm (mean ± standard error, 10 beads at each depth), suggesting that submicron resolution is achievable in the *xy* plane. Further, there was no significant difference in the lateral FWHM between 200 and 500 µm depths, whereas that at 1000 µm was significantly wider. Similarly, the axial FWHMs of the PSF through the light-curable resin were 2.17 ± 0.02 µm at 200 µm, 3.53 ± 0.03 µm at 500 µm, and 5.46 ± 0.09 µm at 1000 µm. In contrast, we performed the experiment without the light-curable resin and found that the lateral and axial FWHM of the PSF at a depth of 200 µm were 511 ± 5 nm and 2.06 ± 0.01 µm, respectively (Supplementary Fig. 5). In the comparison, the axial resolution at 200 µm depth was not significantly different between the with and without the light-curable resin. This indicates that the resolution at 200 µm depth using NIRE method can be as high as that in its absence, although the overall spatial resolution tended to deteriorate (Supplementary Fig. 5e, f). Given that mean cortical thickness is 890 ± 16 µm^44^, these results suggest that the NIRE method produces cranial windows with a sufficient spatial resolution to observe both whole neurons and subcellular structures in upper cortical layers, although as expected the spatial resolution in deep layers could be significantly reduced due to the accumulation of light scattering and optical aberrations.

Finally, we assessed measurement instability due to movement artifacts by comparing the FOV displacement between cranial windows produced using PEO-CYTOP alone, the NIRE method and a glass coverslip with a 4.2 mm diameter, (Fig. 1h–j, Supplementary Fig. 6). Briefly, time-lapse images of SR101-stained astrocytes were acquired, and the correlation coefficient between a reference frame and each individual frame was calculated (*see* Methods). As depicted in Fig. 1h and Supplementary Fig. 6a–d, PEO-CYTOP nanosheets did not suppress FOV displacement because these coverings did not press strongly the cortical surface. In contrast, the NIRE method and the glass coverslip yielded cranial windows, allowing markedly less FOV displacement in both anesthetized and awake mice (Fig. 1i, j and Supplementary Fig. 6e–h). In addition, we quantitatively evaluated motion artifacts using the mean of the correlation coefficients obtained from these measurements (Supplementary Fig. 6m, n). We confirmed that the NIRE method suppresses motion artifacts better than plain PEO-CYTOP nanosheet method, although glass coverslip method was the most stable under all conditions.

Therefore, the light-curable resin was effective in fixing polymer nanosheets to the curved surface of the living brain and suppressing motion artifacts for in vivo imaging.

### Multi-scale imaging of neuronal morphology in vivo

**Fig. 2:**
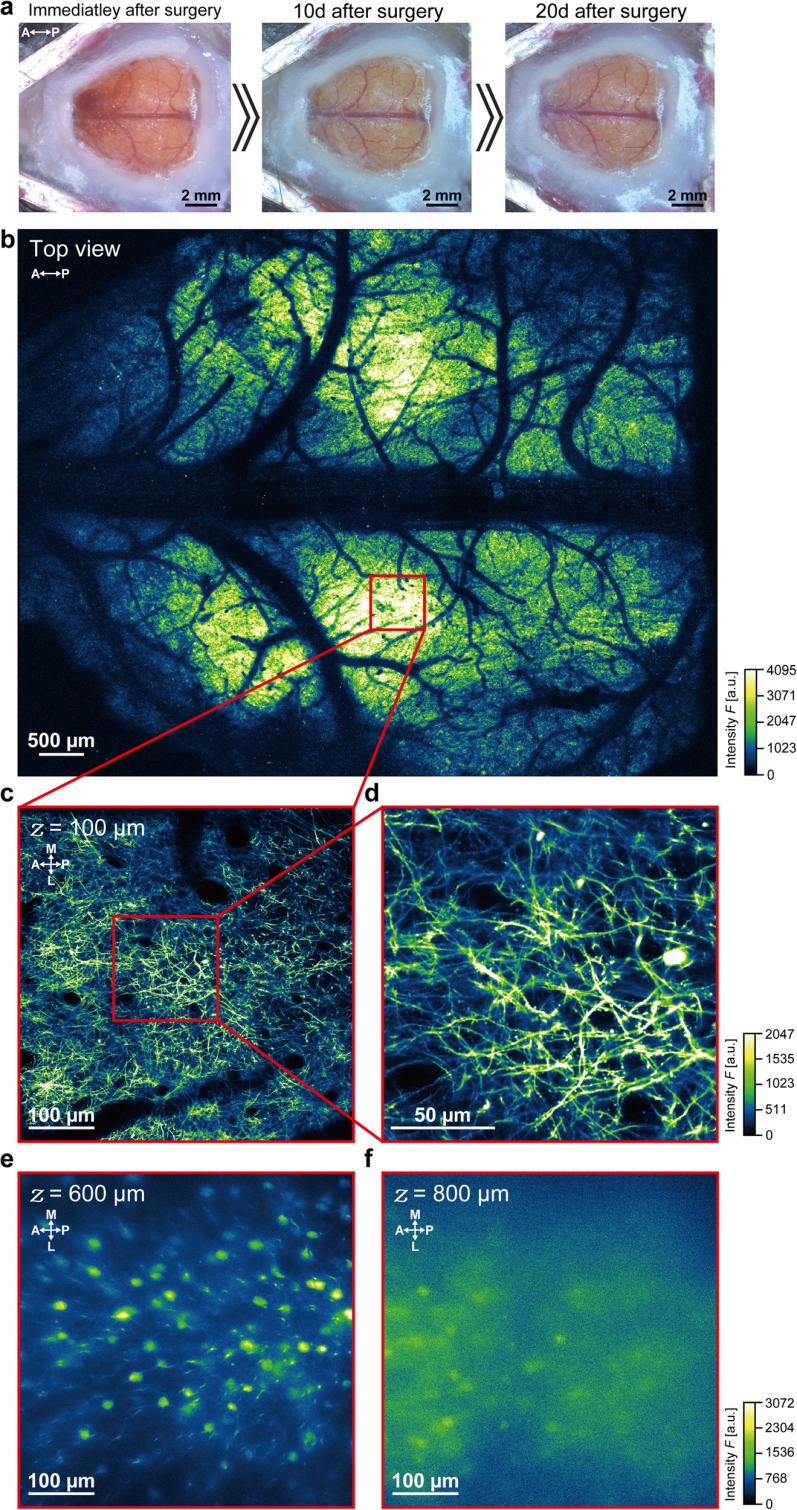
Multi-scale two-photon imaging of Thy1-EYFP-H (H-line) mouse cortex in vivo through a cranial window constructed using the NIRE method. **a** Time-lapse photomicrographs of the large cranial window in an H-line mouse. The directions are indicated as anterior (A), posterior (P). **b** Large field-of-view (~7.8 mm × ~6.0 mm) tiled image derived from the maximum intensity projections of six three-dimensional stacks through the cranial window in **a**. The red box corresponds to the region shown in **c**. The directions are indicated as anterior (A), posterior (P). **c** Cross-sectional *xy*-image of axons and apical dendrites at 100 µm below the cortical surface in the region defined by the red box in **b**. The directions are indicated as anterior (A), posterior (P), medial (M), and lateral (L). **d** Cross-sectional *xy*-image of axons and dendrites with dendritic spines from the region indicated by the red box in **c**. **e**.Cross-sectional *xy*-image of somata and dendrites of neurons at 600 µm below the cortical surface in the region defined by the red box in **b**. The directions are indicated as anterior (A), posterior (P), medial (M), and lateral (L). **f** Cross-sectional *xy*-image of somata of neurons at 800 µm below the cortical surface in the region defined by the red box in **b**. The directions are indicated as anterior (A), posterior (P), medial (M), and lateral (L). Olympus XLFLUOR4X/340 ×4/0.28 NA air-immersion objective lens was used in **b**. Nikon Apo LWD 25×/1.10 NA water-immersion objective lens was used in **c**–**f**.

To evaluate the long-term mechanical and optical stability of these large cranial windows created using the NIRE method, we examined cortical neuron morphology over several weeks in Thy1-EYFP-H mice (expressing enhanced yellow fluorescent protein driven by the Thy1 promoter) (Fig. 2). The transparency of the cranial window was maintained for at least 20 days after surgery (Fig. 2a). Some of the blood vessels imaged earlier can no longer be seen in the photographs taken 20 days after surgery due to reduction in transparency of the cranial window and the reflection from the differently positioned light source. Although bleeding occurred under the dura, this visual occlusion disappeared within 10 days after surgery. Through the large cranial window, images with a broad FOV (~7.8 mm × ~6.0 mm) obtained using a ×4 objective lens (Fig. 2b) revealed widespread fluorescence emission across the parietal cortex, whereas a ×25 objective lens yielded cross-sectional *xy*-images at a depth of 100 µm with clearly distinguishable axons and dendrites (Fig. 2c). Further, dendritic spines could be resolved at this same image plane (Fig. 2d). Although blurry (compared to those taken at 600 µm depth), images of neuronal structures could be taken at 800 µm depth, the deep brain region (Fig. 2e, f). Therefore, the NIRE method could be used for the long-term monitoring of neuronal morphology across multiple regions of the cerebral cortex during maturation, various training regimens, or pathological processes.

### Multi-scale Ca^2+^ imaging in awake mice in vivo

**Fig. 3:**
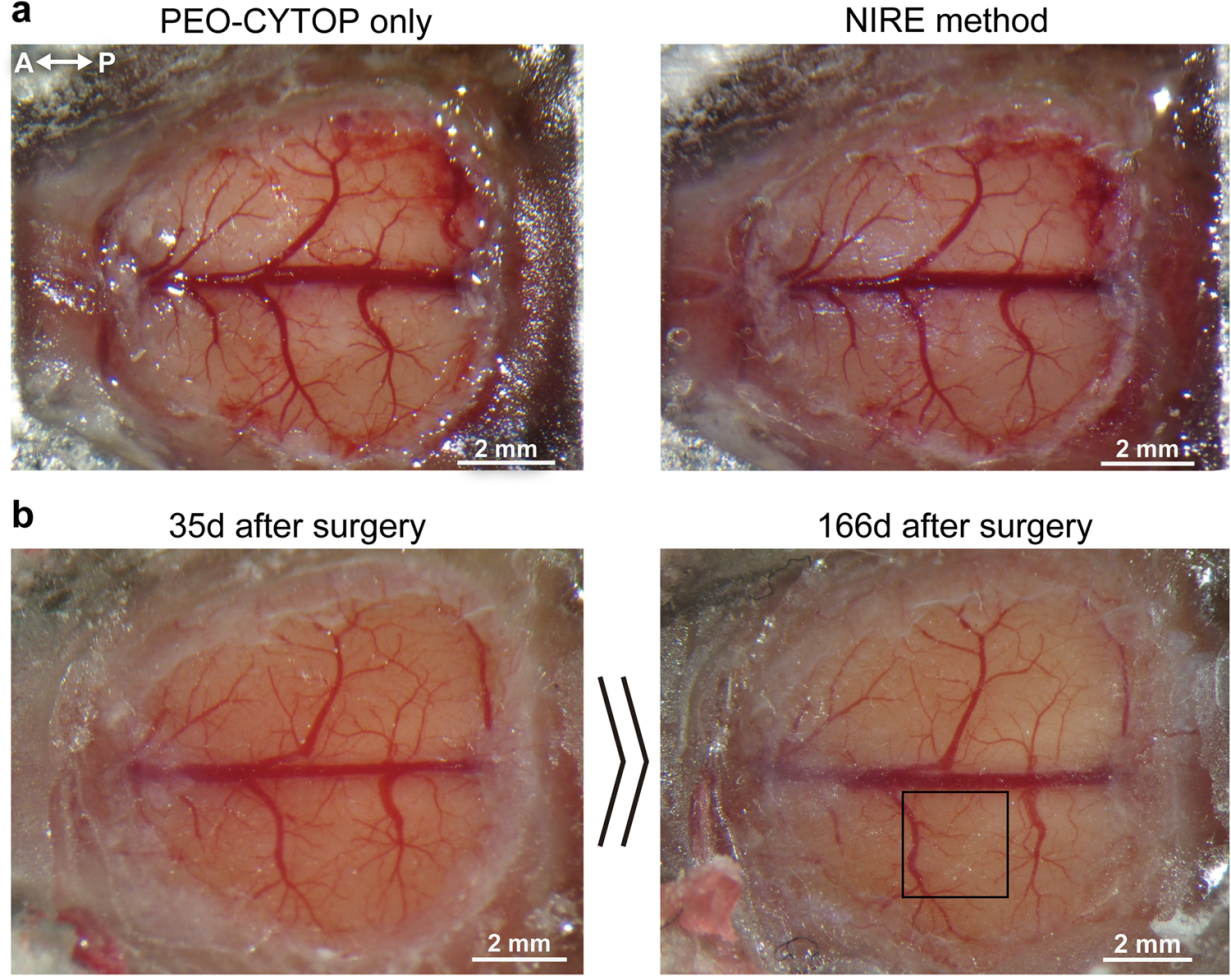
Transparency of a large-scale cranial window produced using the NIRE method. **a** Photomicrographs of a large cranial window before and after fixing the light-curable resin on the PEO-CYTOP nanosheet. The directions are indicated as anterior (A), posterior (P). **b** Photos of the cranial window in **a** 35 days and 166 days after surgery. The black box corresponds to the region in Fig. 4a.

We then evaluated the suitability of these cranial windows for Ca^2+^ Imaging in wide regions of cortex and individual neuron compartments of awake mice. For this purpose, the Ca^2+^ indicator jGCaMP7f was expressed in layer 2/3 of the cerebral cortex by injection of the AAV-DJ-syn-jGCaMP7f vector prior to cranial window creation using the NIRE method (Fig. 3a). We found that the transparency of the cranial window was maintained for over 166 days in three out of four mice (one died the day after the NIRE method probably because of the adverse effect of the surgery) and intracellular calcium measures were obtained (Fig. 3b). While moderate proliferation of blood vessels was observed, these changes did not interfere with in vivo two-photon imaging.

**Fig. 4:**
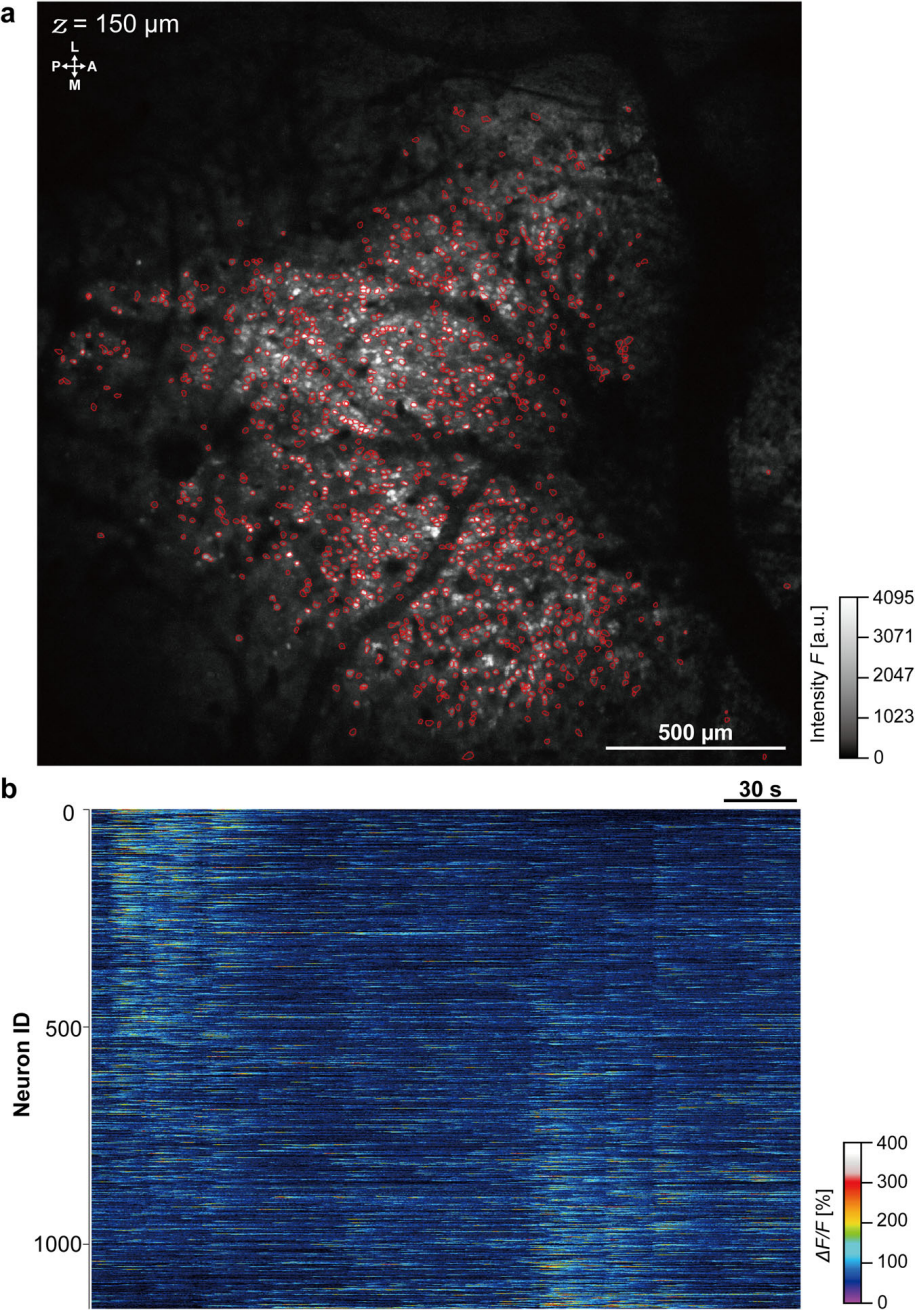
Large-scale fluorometric calcium imaging of neurons through a cranial window produced using the NIRE method. **a** Large field-of-view (~2.1 mm × ~2.1 mm) image produced from the maximum intensity projections of time-lapse Ca^2+^ imaging data acquired over 5 minutes. The red circles demarcate the ROIs, yielding the heatmap of individual neuron ΔF/F values in **b**. The directions are indicated as the anterior (A), posterior (P), medial (M), and lateral (L). **b** Heatmap showing the fluorescence intensity changes in 1158 neurons identified using the CNMF algorithm (*see* Methods). Olympus XLFLUOR4X/340 ×4/0.28 NA air-immersion objective lens was used in **a**.

**Fig. 5:**
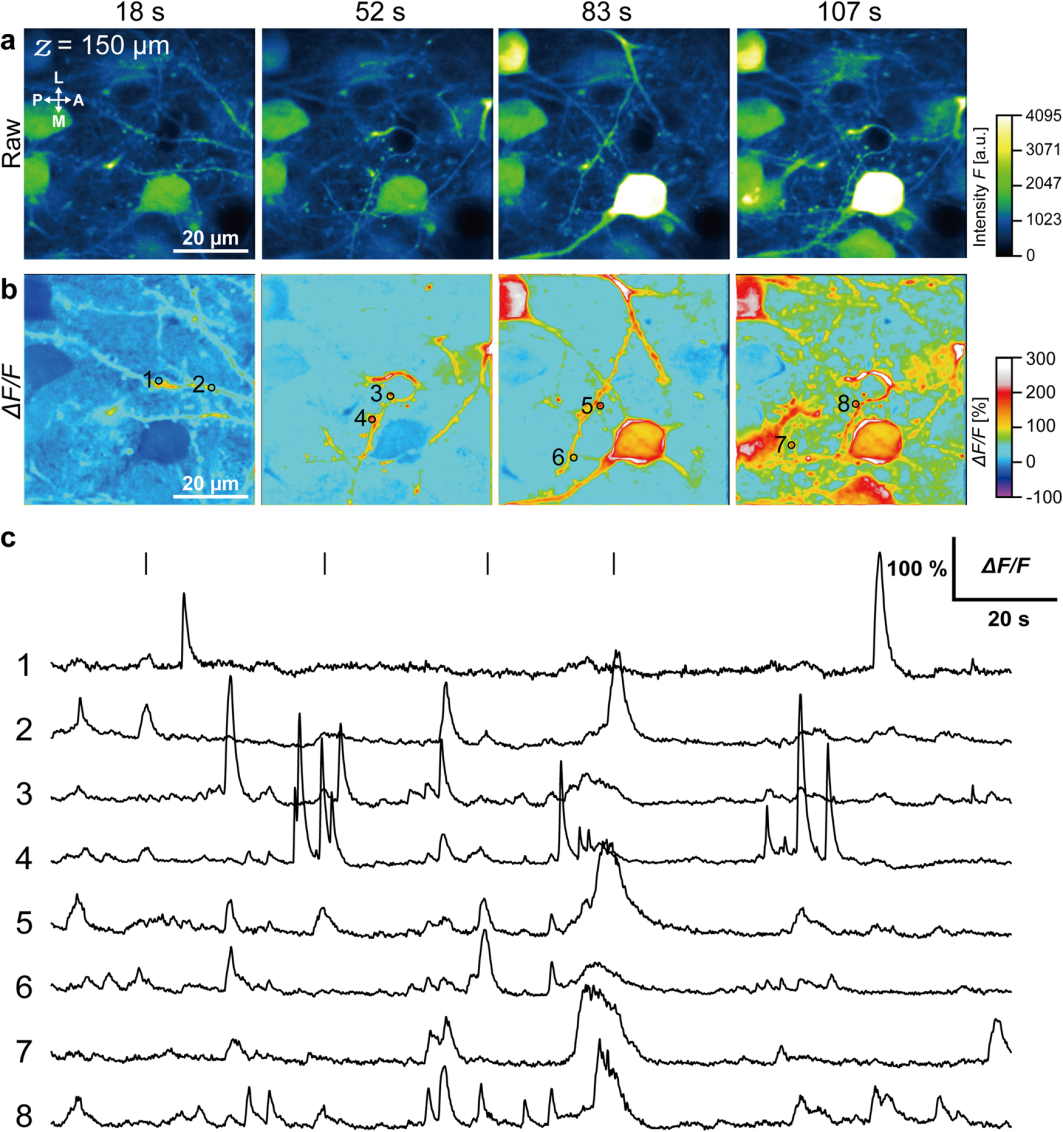
In vivo intracellular calcium imaging of somata, dendrites, and spines through a cranial window produced by the NIRE method. **a** Time-lapse fluorescent images of intracellular Ca^2+^ elevations in the cortex of the same mouse as in Figs. 3 and 4. The acquisition times of the images in **a** and **b** are indicated by the short solid lines in **c**. The directions are indicated as anterior (A), posterior (P), medial (M), and lateral (L). **b** Time-lapse images of fluorescence intensity changes (*ΔF/F*) calculated from the images in **a**. The black circles and numbers represent the ROIs for the fluorescence intensity traces in **c**. **c** *ΔF/F* traces from the ROIs of **b**. The short solid lines indicate the time of **b**. Nikon Apo LWD ×25/1.10 NA water-immersion objective lens was used in **a**, **b**.

Changes in intracellular calcium concentration were measured over a large region of the primary somatosensory cortex ( ~2.1 mm × ~2.1 mm) using a ×4 objective lens and a frame capture rate of 3.8 per second. In total, fluorescence signals from 1158 neurons were extracted over a 5-minute recording period (Fig. 4b, Supplementary Movie 1) using EZcalcium (*see* Methods). To verify the spatial resolution, we also attempted to measure spontaneous Ca^2+^ activity from dendrites and dendric spines at a depth of 150 µm (same awake mouse as in Figs. 3 and 4) using a 25× objective lens (Fig. 5a). Fluorescence intensity changes were clearly revealed by analysis of time-lapse images (Fig. 5b, Supplementary Movie 2) from ROIs on the dendritic spines (Fig. 5c), with peak *ΔF/F* ranging from 85% to 245%. These findings suggest that the NIRE method could allow multi-scale in vivo calcium imaging in the cerebral cortex of awake mice during various sensory stimulation protocols.

### Large-scale imaging from cerebral cortex and cerebellum in vivo

**Fig. 6:**
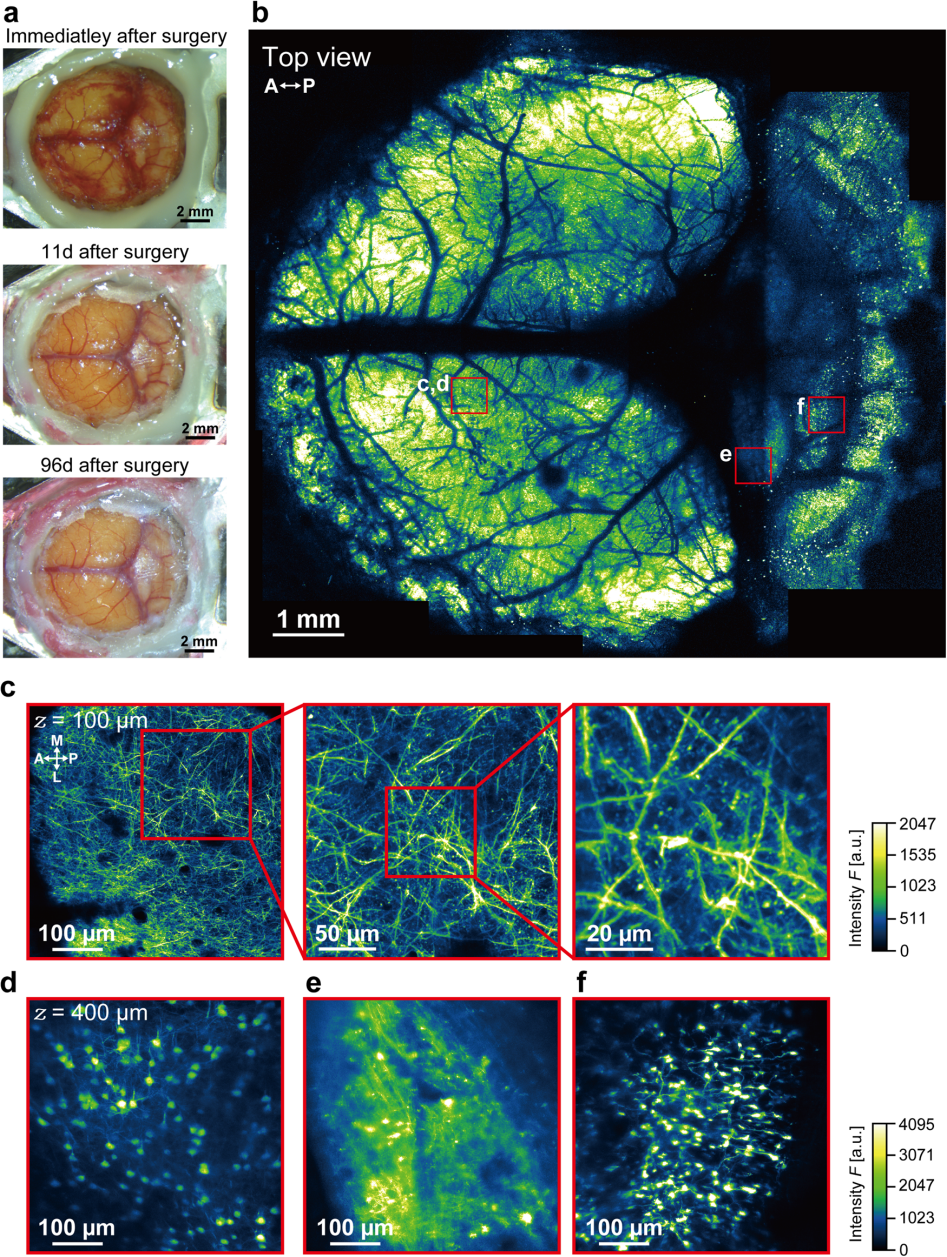
In vivo two-photon imaging through a large cranial window extending from the cerebral cortex to cerebellum. **a** Time-lapse photomicrographs of a large cranial window extending across the cerebral cortex, inferior colliculus, and cerebellum of an H-line mouse. **b** Large field-of-view (~9.7 mm × ~8.8 mm) tiled image derived from the maximum intensity projections of 17 three-dimensional stacks acquired through the cranial window in **a**. The red boxes demarcate the regions shown in **c**–**f**. The directions are indicated as anterior (A), posterior (P). **c** Cross-sectional *xy*-image of axons, apical dendrites, and spines 100 µm below the cortical surface within the red box in **b**. The directions are indicated as anterior (A), posterior (P), medial (M), and lateral (L). **d** Cross-sectional *xy*-image of a soma 450 µm below the cortical surface within the red box in (**b**). **e** Cross-sectional *xy*-image of axons, dendrites, and somata in the inferior colliculus within the red box in **b**. **f** Cross-sectional *xy*-image of cerebellar granular cells within the red box in **b**. Olympus XLFLUOR4X/340 ×4/0.28 NA air-immersion objective lens was used in (**b**). Nikon Apo LWD ×25/1.10 NA water-immersion objective lens was used in **c**–**f**.

To further demonstrate the versatility of the NIRE method for imaging applications beyond the cerebral cortex, we created larger cranial windows extending from the cerebral cortex to the cerebellum in Thy1-EYFP-H mice (Fig. 6a). Again, while bleeding occurred under the dura immediately after surgery, this was cleaned up within 11 days. Through this cranial window, we performed two-photon imaging of multiple regions and constructed three-dimensional stacks of neuronal structures over a ~9.7 mm × ~8.8 mm FOV (Fig. 6b, Supplementary Movie 3). These images revealed the spines, axons, dendrites, and somata of layer I–V neurons in the cerebral cortex (Fig. 6c, d) as well as neurons and neuronal substructures in the inferior colliculus and cerebellum (Fig. 6e, f).

In addition, the long-term monitoring of the cranial window revealed that the transparency was maintained for >6 months (Supplementary Fig. 7). We also measured the fluorescent intensity of neurons in the cerebral cortex and the cerebellum to evaluate the long-term optical properties of the cranial window made using the NIRE method (Supplementary Fig. 8). The fluorescent intensity of neurons in the cerebral cortex did not change at any measured depth (Supplementary Fig. 8a–c, e–g). However, the fluorescent intensity of neurons in the cerebellum (located at the edge of the cranial window) decreased over 6 months (Supplementary Fig. 8d, h). Furthermore, we quantitatively evaluated the brain-region-dependent decay of optical properties of the cranial window (Supplementary Fig. 9). We evaluated the neuronal soma size at different locations under the cranial window (at the center, the edge, and the middle (between the center and the edge)) using the in vivo imaging data shown in Fig. 6, obtained 96 days after surgery. Note that, an air-immersion objective with ×4 magnification and 0.28 NA was used in the evaluation of Supplementary Fig. 9, unlike Supplementary Fig. 5. The lateral FWHMs of the neuronal soma were 10.3 ± 0.4 µm, 10.4 ± 0.3 µm and 10.9 ± 0.4 µm at the center, middle, and edge, respectively (Supplementary Fig. 9k). The axial FWHMs of the neuronal soma were 79 ± 1.4 µm, 86 ± 2.2 µm, and 113 ± 2.6 µm at the center, middle, and edge, respectively (Supplementary Fig. 9l). Thus, although the lateral resolution was not degraded significantly in all regions, we can see that the axial resolution is degraded in the edge region, at a radial distance of approximately 3 mm from the center of the cranial window (Supplementary Fig. 9k, l). This indicates that the NIRE method realizes long-term imaging in both the cerebral cortex and the cerebellum up to 6 months after surgery; however, the spatial resolution is degraded at the edge of the cranial window >6 months after surgery.

In conclusion, the NIRE method expands the simultaneous/segregated in vivo brain imaging range and widens it to encompass the cerebral cortex and cerebellum, helping visualize their neuronal morphology and function.

## Discussion

In this study, we developed a new method for producing large cranial windows suitable for long-term, multi-scale, two-photon imaging of the living brain utilizing PEO-CYTOP nanosheets and light-curable resin. Using this NIRE method, we successfully obtained high-resolution images of neuron somata, dendrites, and spines in all layers of the cerebral cortex, as well as fluorometric measurements of intracellular calcium in awake mice. In addition to widely distributed cortical neurons, we obtained images of granule neurons in cerebellum, underscoring the utility of this method for in vivo imaging of neural network activity. Further, we demonstrated that these images can be obtained in the awake state without substantial motion artifacts.

In our previous study, we found that PEO-CYTOP nanosheets could be used to fabricate cranial windows that conformed to the complex topography of the cortical surface, thereby reducing mechanical stress and mitigating potential disturbances in cerebrospinal fluid (CSF) and blood flow. However, these PEO-CYTOP nanosheets did not suppress the motion artifacts caused by respiration and body movements in awake mice. To effectively suppress motion artifacts while preserving vital physiological functions, in this study we coated PEO-CYTOP nanosheets with a light-curable resin (the NIRE method, Fig. 1, Supplementary Fig. 6).

Moreover, the chemically inert and nonbiodegradable fluoropolymer CYTOP prevented the light-curable resin from contacting the brain surface, thereby permitting long-term imaging without induction of neurotoxicity and neuroinflammation (Fig. 1e–g)^45^. In addition, PEO is commonly used as a coating material to suppress inflammatory reactions^46^. The PEO-CYTOP nanosheet also suppresses inflammation^34^. However, long-term observation through PEO-CYTOP nanosheets alone is difficult because the PEO-CYTOP nanosheet is not fixed and floats with intracranial pressure changes and brain edema^34^. With NIRE method, the light-curable resin tightly fixes the nanosheets to the brain surface and eliminates gaps where dura mater could regrow in long term. Thus, once established in an animal, the cranial widow created using NIRE method enables long-term (≥5 months) broad FOV in vivo imaging with subcellular resolution due to the substantial mechanical and optical stability of the nanosheet–resin combination (Fig. 3–5). Hence, we suggest that the chronic cranial windows made by the NIRE method are suitable for the examination of neuroplasticity at the network, circuit, and cellular levels during maturation and behavioral training as well as neurodegeneration during disease progression. For example, this method may further facilitate investigations into the mechanisms of neural population coding underlying associative memory^47^ and neural circuit remodeling accompanied by chronic pain^48^, which have been elucidated by the advantages of longitudinal in vivo two-photon microscopy.

However, observable period is limited in the NIRE method due to various reasons. Firstly, protecting the transparency of the light-curable resin surface of the cranial window (when the mouse is housed in its cage (*see* Methods) is riddled with issues of fine debris adherence, and the detrimental cleaning procedure which degrades the transparency. Secondly, adhesion of immune cells to the internal surface of the cranial window, regrowth of dura mater, along with the gradual accumulation of bone and connective tissue, result in the clouding of the cranial window^30,49,50^. We surmise that the observable period is limited by these factors (existing on inside and outside surfaces of the cranial window) that reduce transparency. Moreover, during the long-term monitoring of cranial windows—created using the glass coverslip and the NIRE methods—we found that the opacity mainly spreads from the edge to the center (Supplementary Figs. 7 and 8). Thus, we conclude that the large cranial windows created using the NIRE method retain transparency longer than conventional small cranial windows due to their effectively large central area, despite the gradual decrease in transparency along their edges (Supplementary Fig. 8).

In using the light-curable resin in the NIRE method, we reasoned that the heat used for curing would damage brain tissue. To circumvent this, we employed intermittent irradiation and succeeded in curing the resin at a temperature that is not harmful to the living tissue (Supplementary Fig. 1). Although it is possible to cure the light-curable resin using low-power continuous irradiation, the procedure time and the stress on the mouse increases; curing with low-power continuous irradiation requires longer time than that with high-power intermittent irradiation.

Moreover, steps involved in cranial window creation lower the brain temperature which leads to abnormal neural activity^51^. The manufacture-specified thermal conductivity of the resin used here (NOA83H) is 0.17 W m^−1^ K^−1^, which is lower than the thermal conductivities of glass coverslips (1.05 W m^−1^ K^−1^) and bone (~0.68 W m^−1^ K^−1^)^52^. Therefore, unlike conventional cranial windows made using glass coverslips, those created using the NIRE method can reduce the drop in brain temperature and its consequent adverse effects on neural activity.

Moreover, craniectomy for cranial windows inevitably alters the brain temperature and flow of blood and CSF, which may adversely affect neural activity and brain function. The NIRE method circumvents these issues by creating a cranial window that conforms to the shape of the brain surface and advantageously preserves the shape of blood vessels and the CSF flow path. Hence, we conclude that compared to other methods used to create cranial windows (in which the materials are fixed before the craniectomy leading to compressed blood vessels and CSF flow path), the NIRE method keeps the brain tissue healthier.

Using the NIRE method, cranial windows up to 9 mm in diameter were produced, substantially larger than the 2–5-mm diameter cranial windows produced using a glass coverslip^29,30^. Several novel techniques have been proposed to broaden the FOV through cranial windows in living mice^31–33^, including a chemical skull clearing method^53–55^. However, thick skull and many blood vessels makes it difficult to apply these methods to observe the cerebellum and midbrain. Further, the PEO-CYTOP nanosheet in the NIRE method suppresses the bleeding from the brain surface after the craniectomy because of its high adhesive strength^34^. This hemostatic effect of the PEO-CYTOP nanosheet facilitates the creation of large cranial windows by preventing operational and postoperational bleeding (unlike in conventional cranial window creation methods). Additionally, the highly curved surfaces of cerebellum and midbrain make it difficult to obtain sufficient optical resolution using conventional imaging methods. In contrast, our NIRE method facilitates the production of cranial windows extending across the cerebral cortex, midbrain, and cerebellum for both large FOV and cellular/subcellular imaging in vivo (Fig. 6). Therefore, this approach may facilitate the elucidation of higher brain functions dependent on the coordinated activity of widely distributed brain regions.

On the other hand, the light-curable resin used in the NIRE method was supposed to be an optical disturbance, particularly at a deep region of samples, compared to PEO-CYTOP nanosheets alone^34^ (Supplementary Figs. 4 and 5). The reduced spatial resolution may be due to the high refractive index (~1.56) of this compound, resulting in spherical aberrations, and nonuniform surface, leading to coma and astigmatism. These aberrations also occurred in experiments using fluorescent beads embedded in agarose gel, which deteriorated the spatial resolution and made observations difficult at the diffraction limit. In in vivo experiments, large cranial windows exhibit unavoidable optical aberrations due to curvature of the brain surface (Supplementary Fig. 9). The large cranial window region covering cerebral cortex allowed imaging to a depth of ~800 µm (Fig. 2e). Whereas the that covering the cerebellum allowed clear visualization of neuronal structures only to a depth of approximately 400 µm (Fig. 6f). Results from in vitro and in vivo experiments lead us to think that the observable depth in the NIRE method is limited by the cumulative effect of light scattering and optical aberrations. In future studies, we plan to address these issues by employing light-curable resins with lower refractive indices to reduce the mismatch with brain tissue. However, the use of light-curing resins with a lower refractive index may adversely affect the mice due to the higher energy required to cure them. Moreover, the integration of the NIRE method with recently developed adaptive optics techniques^56–60^ could compensate for optical aberrations, thereby enabling even higher resolution in vivo imaging.

The other issue associated with imaging using large cranial windows created by NIRE method is the capture of fluorescence from neurons in multiple layers as a single-plane image due to the preserved curvature of the brain. This problem can be solved by adding an optical device to the imaging set-up that allows capture of neuronal activities from a single optically delineated layer. Specifically, devices (such as a piezoelectric device or an electrically tunable lens) that can image multiple planes along the Z-axis at high speed would be effective in measuring neuronal activities in a single cortical layer through the large cranial window.

Several previous studies examining the neural basis of social behavior have successfully obtained optical measurements of neural activity in freely moving animals^21,61–65^. However, large-scale imaging of neural activity in freely moving mice has generally been performed through the skull, which diminishes the signal-to-noise ratio, especially from deep cortical neurons. In contrast, cranial windows produced using the NIRE method may yield sufficient signal-to-noise to enable in vivo imaging of deep brain regions. Additionally, our method can be integrated with techniques such as ectopic expression of tracers or functional proteins using AAVs, direct intracerebral administration of drugs, and electrophysiological recording.

Since it can effectively prevent motion artifacts without causing mechanical stress and inflammation, the NIRE method can be used for long-term in vivo imaging of various other organs. However, a scaffold device (comparable to those used in previous studies)^66–68^ would be required to realize in vivo imaging of organs that lack a natural scaffold to fix the sealant (unlike brain, which has skull) while using the NIRE method. Additionally, a protective device similar to that used here (for brain) may also be needed to protect the transparency of the light-curable resin window, which deteriorates by contact with the mouse body and debris.

In conclusion, the NIRE method enables in vivo imaging of neuronal structure and intracellular Ca^2+^ changes on scales ranging from single dendritic spines to several hundred widely distributed cortical neurons over weeks in the awake mouse. This method also facilitates the creation of cranial window large enough to view the cerebral cortex, midbrain, and cerebellum simultaneous for neural network studies. Collectively, these properties may facilitate the elucidation of neural network activities and neuroplastic changes underlying higher brain functions as well as disease processes in model animals.

## Methods

### Animals

Immunostaining and intracellular calcium imaging experiments were conducted on adult wild-type C57BL/6 J mice (males and females over 8 weeks of age), whereas analyses of neuronal morphology were conducted on Thy1-EYFP-H (H-line) transgenic mice^69^. All mice were housed under a 12 h/12 h light/dark cycle. These genetic recombination experiments were approved by the Safety Committee on Genetic Recombination Experiments of the National Institutes of Natural Sciences. The facility used for the care and management of laboratory animals was approved by the Institutional Animal Care and Use Committee of NIPS and recombinant DNA Experiments Safety Committee of the National Institute for Physiological Sciences.

### Nanosheet

PEO-CYTOP nanosheets with a thickness of ∼130 nm that exhibited a water retention effect and a hydrophilized adhesive surface were prepared as previously described^34^. Briefly, perfluoro(1-butenyl vinyl ether) polymer (CYTOP, CTX-809SP, AGC Inc., Japan) was dissolved in perfluorotributylamine at 30 mg/mL for spin coating. Silicon wafers layered with 200 nm silicon oxide were used as substrates. First, poly(vinyl alcohol) (PVA, Mw: 22,000, Kanto Chemical Co., Inc., Japan) dissolved in water at 10 mg/mL was spin-coated at 4000 rpm for 20 s on the substrate as a sacrificial layer. The CYTOP solution was then spin-coated onto the PVA-coated substrate, followed by a spin-coating of a 10:1 Sylgard 184 silicone elastomer mixture. After curing at 80 °C for 2 h and exposure to oxygen plasma, a hydrophilic surface was created using 2-(methoxy(polyethyleneoxy)propyl) trichlorosilane (PEO-silane, Gelest, Inc., PA, USA) dissolved in toluene. The nanosheets detached from the substrate as the sacrificial layer dissolved which were then re-supported on a nonwoven fabric. Nanosheets transferred on a nonwoven fabric substrate with the hydrophilic side facing outward were stuck on living mice brains. The composition and properties of the PEO-CYTOP nanosheet created here were similar to that reported earlier^34^. The light transmittance of the PEO-CYTOP nanosheets supported on the quartz glass was measured using a UV-Vis-NIR spectrophotometer (V-670, JASCO Corp., Japan) at a wavelength of 400–1000 nm.

### Craniectomy using PEO-CYTOP nanosheet

Craniectomy was performed as previously described^34,70^. Briefly, anesthesia was induced by 3% isoflurane and maintained during surgery using 1.5% isoflurane. Body temperature was maintained throughout the operation using a disposable heating pad. GLYCEOL (TAIYO Pharma, Japan) was administered 15 minutes before surgery via intraperitoneal injection (15 µL/g) to reduce intracranial pressure, thereby loosening the dura and making the cortical surface as flat as possible. The hair and skin were then removed to expose the cranial skull, and the chosen region removed using a dental drill and tweezers. Achieving large-scale cranial windows with long-term transparency requires extreme skills in performing craniectomy. Severe injuries to brain tissue, such as those caused by drill piercing the brain tissue, cause proliferative spread of connective tissue from the damaged area to the entire surface of the cranial window, making observations at the single-cell resolution difficult.

Following craniectomy, 25 µM Sulforhodamine 101 (SR101) was applied onto the brain surface for 5 minutes to stain astrocytes. In some experiments, an adeno-associated virus (AAV) expressing a fluorescent calcium probe (adeno-AAV-DJ-syn-jGCaMP7f, Addgene plasmid #104488; http://n2t.net/addgene:104488; RRID: Addgene_104488)^71^ was pressure injected (500 nL of 6.16 × 10^9^ vector genomes/mL at 10 psi) 150-µm below the dura using a glass micropipette. After washing away dust and blood with saline, the PEO-CYTOP nanosheet supported by a nonwoven fabric was placed onto the exposed brain surface. The edges of the PEO-CYTOP nanosheet were glued with light-curable resin to prevent immersion solution from contacting brain tissue. For in vivo imaging of awake mice, an aluminum head plate described previously^70^ was fixed to the mouse skull with super-bond (Sun Medical, Japan) and connected by a screw to a 1-cm thick stage weighing over 1 kg.

### Lamination of PEO-CYTOP nanosheets with light-curable resin

After sealing the cranial hole with a PEO-CYTOP nanosheet, light-curable resin (NOA83H, Norland Products Inc., USA) was dripped on the PEO-CYTOP nanosheet. The light-curable resin NOA83H has high transparency (Supplementary Fig. 3) and enough hardness (85D Shore) to fully fix the shape of the PEO-CYTOP nanosheets for long term. The light-curable resin on the PEO-CYTOP nanosheet was fixed using a manual UV irradiator (JAXMAN, China) or programmable UV irradiator (maximum radiant power of 200 mW) consisting of an LED control board (ADULEDB, Bit Trade One, Japan) and UV-LED (OSV1XME3E1S, OptoSupply, Hong Kong) (Supplementary Fig. 1). The optimal irradiation conditions to fix the resin without generating excessive heat were 365 nm excitation for 2 seconds once every 30 seconds over 3 minutes and then 10 mw for 10 seconds every 30 seconds over 7 minutes. The light-curable resin layer ranged in thickness from 100 to 400 µm.

To protect long-term transparency of the cranial window when the mice were housed in their cages, we gently placed an appropriately sized mixing paper (Mixing paper No.10, Tokuyama Dental Corp., Japan) on the cranial window and sealed the cranial window with a vinyl tape. The mixing paper avoids direct contact between the tape and the surface of the cranial window. This protection protects the cranial window from scratching from mouse limbs. The tape must be replaced regularly to prevent accidental removal. It was reapplied approximately once a month. The debris adhered to the surface of the cranial window was either sucked with a suction pump or cleaned with water. Stubborn debris was rubbed off with a cotton swab. UV irradiation pattern for curing was selected after studying the temperature changes initiated in the light-curable resin by the irradiation; the temperature before, during, and after UV irradiation were recorded using the digital thermometer electrodes (TR-71nw, T&D Corporation., Japan) inserted in the light-curable resin placed in a 35 mm petri dish. The light transmittance of the light-curable resin “NOA83H” was measured using a fluorescence and absorbance spectrometer, (Duetta, HORIBA, Ltd., Japan) at a wavelength of 400–1000 nm.

### Image acquisition

All fluorescence images were obtained using a two-photon laser microscopy customized for in vivo imaging (A1R-MP+, Nikon). We summarized the parameters for in vivo imaging shown in Supplementary Table 1. The Olympus XLFLUOR4X/340 4×/0.28 NA air-immersion objective lens was used to obtain images with a broad field of view (FOV) (e.g., Figs. 2, 4 and 6, Supplementary Fig. 9), whereas a Nikon Apo LWD ×25/1.10 NA water-immersion objective lens was used for imaging of single neurons within cortex (e.g., Figs. 2, 5 and 6, Supplementary Fig. 8). The correction collar of the objective lens was adjusted manually by checking the brightness of the images. A Ti:Sapphire laser (MaiTai eHP DeepSee, Spectra Physics) was used as an excitation light source. EYFP images were acquired using an excitation wavelength of 950 nm and jGCaMP7f images at 960 nm. Image stacks were acquired using z-steps of 5 µm (Fig. 2) or 15 µm (Fig. 6, Supplementary Fig. 8). All fluorescence emission signals shorter than 690 nm were detected using non-descanned detectors (NDDs) equipped with GaAsP photomultiplier tubes at 0.5 frame per second (fps) for EYFP and 3.8 or 7.5 fps for jGCaMP7f. The NDD sensitivity and laser power were adjusted according to individual experimental requirements. The fluorescence signal was separated using two dichroic mirrors (560-nm dichroic mirror for yellow–green beads, EYFP, and jGCaMP7f, and 593-nm dichroic mirror for SR101).

The results shown in Figs. 2 and 6, and Supplementary Figs. 6, 8, and 9 were obtained from a mouse under anesthesia, whereas those in Figs. 4 and 5, and Supplementary Fig. 6 were obtained from a mouse in awake condition. Body temperature was maintained during imaging using a disposable heating pad.

To quantify the spatial resolution in the NIRE method (Supplementary Figs. 4 and 5), yellow–green beads (200 nm diameter, Invitrogen) were embedded at varying depths in a 1% agarose gel (1:100, v/v) in a 35 mm plastic dish. Then, samples were prepared with or without covering the surface of the gel with a light-curable resin film (approximately 150 µm thick, similar to the in vivo conditions). To achieve the fluorescent images of yellow–green beads, we put the sample horizontally under a Nikon Apo LWD ×25/1.10 NA water-immersion objective lens for two-photon microscopy (A1R-MP+, Nikon, Japan). Image stacks were acquired with z-steps of 0.2 µm. This imaging protocol yielded a pixel size of 50 nm. Full width at half maximum (FWHM) values of the PSFs were then calculated by fitting the fluorescence intensity profiles around the central intensity to a Gaussian function using ImageJ.

The optical properties were evaluated by in vivo imaging, as represented in (Supplementary Figs. 8 and 9). Two-photon imaging of the corresponding fields of view and time series were done using the same laser power and detector sensitivity.

### Immunostaining

Mouse brains were perfusion-fixed with 4.0% paraformaldehyde (PFA) 4 weeks after cranial window surgery over the primary visual cortex (Fig. 1e, f, Supplementary Fig. 2a, b). Brains were removed, fixed overnight in 4% PFA, and cut into 50-μm thick coronal sections using a vibratome (7000smz; Campden Instruments, Leicestershire, UK). Slices were then incubated in 10% blocking solution (NACALAI TESQUE, INC., Japan) containing 0.2% Triton X-100 for 30 minutes at room temperature, followed by incubation with a primary antibody against glial fibrillary acidic protein (GFAP, 1:1,000, FUJIFILM Wako Pure Chemical Corporation, Japan) overnight (~16 h) at 4°C. After washing with 0.1% Tween 20 in PBS, slices were incubated with secondary antibody (1:500; Alexa Fluor 594 donkey anti-mouse IgG; Thermo Fisher Scientific, Waltham, MA, USA) for 4 h at room temperature. Nuclei were counterstained with 1:2000 Hoechst 33258 (FUJIFILM Wako Pure Chemical, Japan). Finally, stained slices were examined using a confocal microscope (Leica TCS SP8 STED 3X FALCON, Leica, USA) with an HC PL APO CS2 ×10/0.40 NA dry objective lens. Mean Hoechst 33258 and GFAP fluorescence emission levels were measured at 15 ROIs from three mice in each condition using ImageJ and normalized per 300 × 300 µm^2^.

### Analysis of FOV displacement

FOV displacement was assessed by calculating a correlation coefficient (Supplementary Fig. 6) in MATLAB (2019b, The MathWorks, Inc., USA) using the following steps: (i) generating a reference frame by calculating the median of all individual frames, (ii) calculating a correlation coefficient between this reference frame and each individual frame, (iii) generating a new reference frame from the median of 10 frames yielding the strongest correlation coefficient in step (ii), and (iv) calculating the correlation coefficient between this new reference frame and each individual frame. Finally, motion artifacts were evaluated using the correlation coefficients obtained from these measurements and the Welch’s *t* test with Bonferroni correction.

### The analysis of Ca^2+^ imaging

The intracellular Ca^2+^ imaging data acquired in this study (Figs. 4 and 5) were processed using the ECC image alignment algorithm^72^. In Fig. 4, fluorescent signals induced by changes in intracellular Ca^2+^ (*ΔF/F*) were segmented and extracted from time-lapse images using EZcalcium, an open-source MATLAB application (2021a) designed for the analysis of large-scale calcium imaging datasets with ROI detection and refinement functions^73^. Alternatively, the Ca^2+^ imaging data shown in Fig. 5 were analyzed using NIS element software (Nikon). Briefly, the software time measurement tool was used to extract the mean fluorescence emission within each ROI at each time point, and the median fluorescence intensity was used as the baseline value to calculate the relative fluorescence change (*ΔF/F)*.

### Statistics and reproducibility

Images were analyzed using MATLAB, Fiji ImageJ, and NIS elements. Graphs and charts were made by MATLAB (2021a) and Microsoft Excel. The degree of neuroinflammation induced by the light-curable resin with and without the addition of the nanosheet was evaluated by counting the proportion of GFAP-positive astrocytes to total Hoechst 33258 stained nuclei and evaluated by using Welch’s *t* test with and without Bonferroni correction (Fig. 1g, Supplementary Fig. 2c). In addition, the spatial resolution values (point-spread functions) of yellow–green fluorescent beads at different depths in agarose (Supplementary Figs. 4 and 5) and the soma size of the neurons (Supplementary Fig. 9) were compared by Welch’s *t* test with Bonferroni correction. In evaluations of motion artifacts, the correlation coefficients between the reference frame and all frames in each condition were compared using Welch’s *t* test with Bonferroni correction (Supplementary Fig. 6).

### Reporting summary

Further information on research design is available in the Nature Portfolio Reporting Summary linked to this article.

### Supplementary information

Supplementary Information

Description of Supplementary Materials

Supplementary Movie 1

Supplementary Movie 2

Supplementary Movie 3

Supplementary Data 1

Reporting Summary

## Data Availability

Source data underlying the graphs and charts are provided in Supplementary Data 1. Other datasets supporting the current study are available from the corresponding author on reasonable requests.
