# Supplementary material for: Large-scale cranial window for in vivo mouse brain imaging utilizing fluoropolymer nanosheet and light-curable resin
Source: Commun Biol. 2024 Mar 4;7:232. doi: 10.1038/s42003-024-05865-8 (PMC10912766; doi:10.1038/s42003-024-05865-8)
Supplement: Supplementary file 1 — Supplementary Information [file 42003_2024_5865_MOESM1_ESM.pdf]

Supplementary Information

Large-scale cranial window for *in vivo* mouse brain imaging utilizing fluoropolymer nanosheet and light-curable resin

Authors and Affiliations

Taiga Takahashi[1,2,3,4], Hong Zhang[5,6], Masakazu Agetsuma[7,8], Junichi Nabekura[3,7], Kohei Otomo[1,2,3,9], Yosuke Okamura[5,10,11], Tomomi Nemoto[1,2,3] *

1. Division of Biophotonics, National Institute for Physiological Sciences, National Institutes of Natural Sciences, Higashiyama 5-1, Myodaiji, Okazaki, Aichi, 444-8787, Japan;
2. Biophotonics Research Group, Exploratory Research Center on Life and Living Systems (ExCELLS), National Institutes of Natural Sciences, Higashiyama 5-1, Myodaiji, Okazaki, Aichi, 444-8787, Japan;
3. School of Life Science, The Graduate University for Advanced Studies (SOKENDAI), Higashiyama 5-1, Myodaiji, Okazaki, Aichi, 444-8787, Japan;
4. Department of Medical and Robotic Engineering Design, Faculty of Advanced Engineering, Tokyo University of Science, 6-3-1 Niijuku, Katsushika, Tokyo 125-8585, Japan;
5. Micro/Nano Technology Center, Tokai University, 4-1-1 Kitakaname, Hiratsuka, Kanagawa 259-1292, Japan;
6. School of Chemical Engineering and Technology, Tianjin University, 135 Yaguan Road, Jinnan District, Tianjin 300350, China;
7. Division of Homeostatic Development, National Institute for Physiological Sciences,

National Institutes of Natural Sciences, Okazaki, 444-8585, Japan;

8. Quantum Regenerative and Biomedical Engineering Team, Institute for Quantum Life Science, Anagawa 4-9-1, Chiba Inage-ku, Chiba 263-8555, Japan;

9. Department of Biochemistry and Systems Biomedicine, Graduate School of Medicine, Juntendo University, 2-1-1 Hongo, Bunkyo-ku, Tokyo 113-8421, Japan;

10. Department of Applied Chemistry, School of Engineering, Tokai University, 4-1-1 Kitakaname, Hiratsuka, Kanagawa 259-1292, Japan;

11. Course of Applied Science, Graduate School of Engineering, Tokai University, 4-1-1 Kitakaname, Hiratsuka, Kanagawa 259-1292, Japan;

Correspondence author and Lead Contact: Tomomi Nemoto (tn@nips.ac.jp)

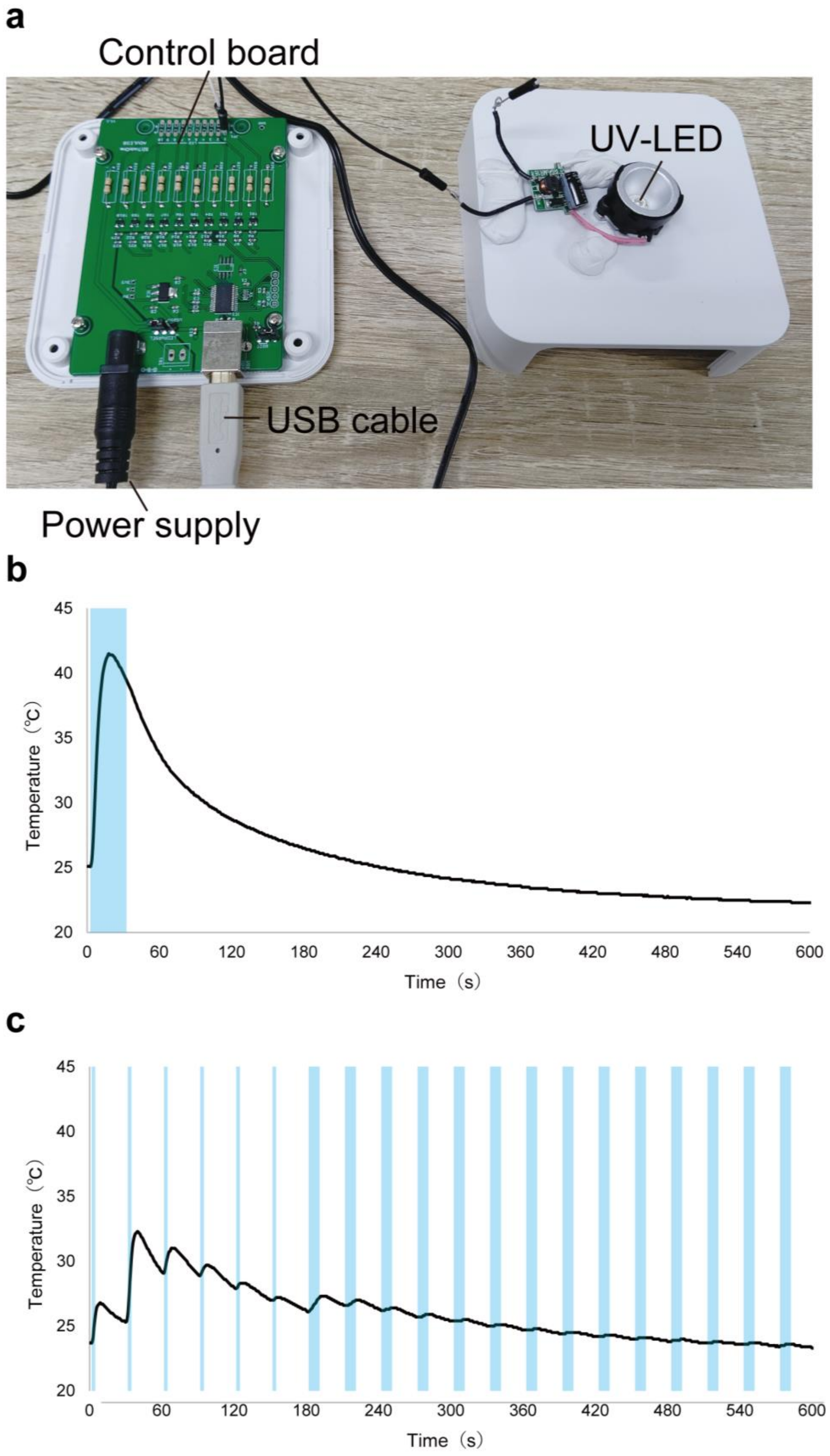
a
Control board
UV-LED
USB cable
Power supply
b
Temperature (°C)
Time (s)
c
Temperature (°C)
Time (s)

Supplementary Figure 1. Time and power programmable UV irradiation system.

a. Photograph of the handmade programmable UV irradiator.

b. Temperature changes over 30 seconds of continuous UV irradiation to fix the light-curable resin. The blue bar indicates the irradiation ON periods.

c. Temperature changes due to intermittent UV irradiation. The light-curable resin was irradiated at intervals of 2 seconds every 30 seconds for 3 minutes and then for 10 seconds every 30 seconds for 7 minutes. Blue bars indicated irradiation ON periods.

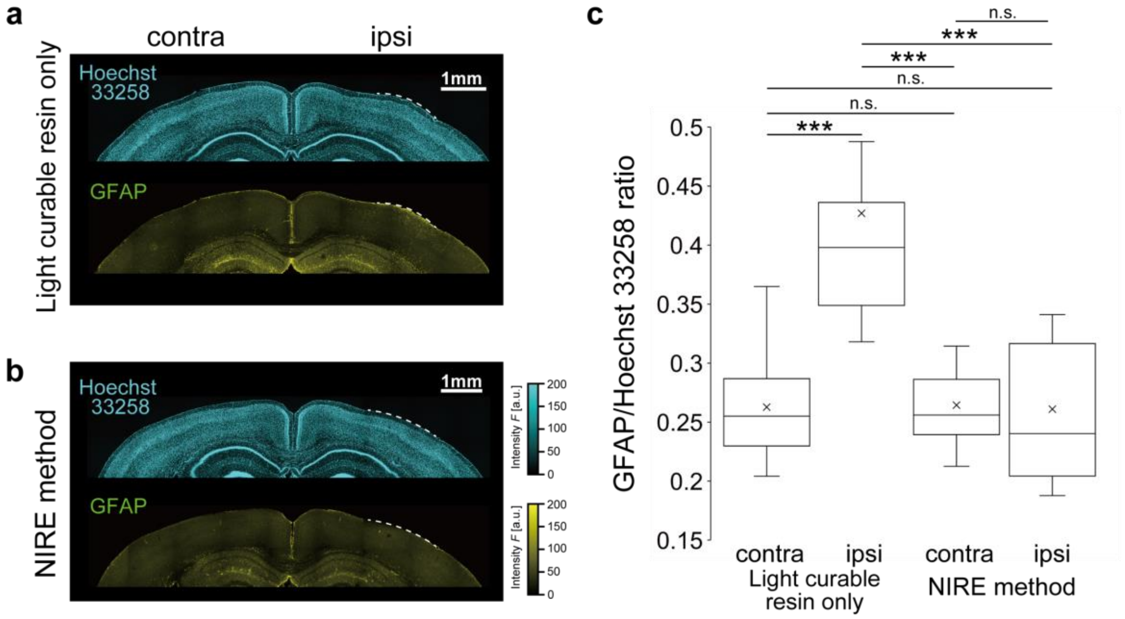

Supplementary Figure 2. Evaluation of neuroinflammation caused by the NIRE method.

a. Immunostaining of astrocytes using anti-GFAP and nuclear counterstaining in a brain slice obtained 4 weeks after craniectomy and covering with light-curable resin only (without a PEO-CYTOP nanosheet). The white dashed line indicates the region where the skull was removed and the resin was fixed.

b. Immunostaining of astrocytes using anti-GFAP and nuclear counterstaining in a brain slice obtained 4 weeks after craniectomy and the NIRE method. The white dashed line indicates the region where the skull was removed and the resin was fixed.

c. Box plots showing the ratio of mean GFAP to Hoechst 33258 signal per 300 × 300 μm^2 in the ipsilateral region and contralateral region (15 ROIs from three mice in each condition). ***$p < 0.005$ by Welch's t-test with Bonferroni correction.

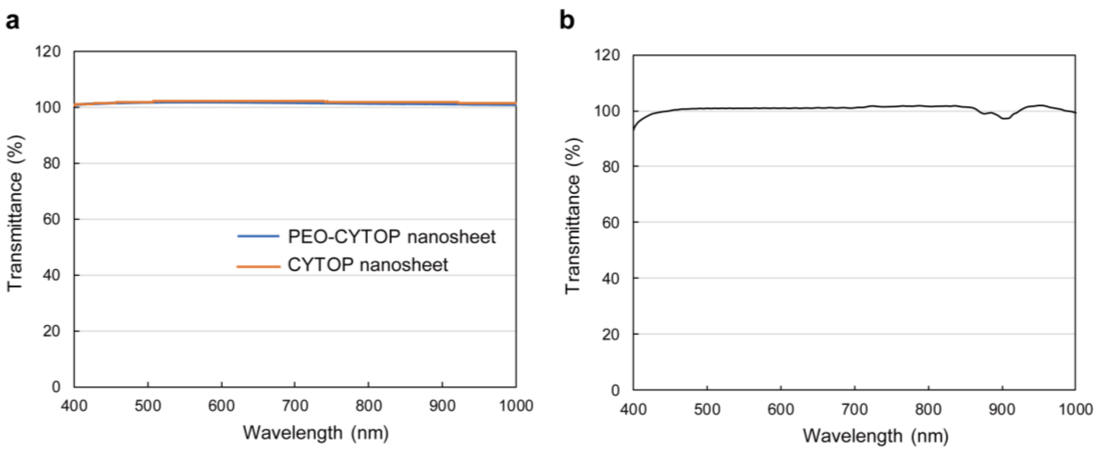

Supplementary Figure 3. Transmittance of the PEO-CYTOP nanosheets and the light curable resin used in the NIRE method

a. Transmittance in the wavelength from 400 to 1000 nm of the CYTOP and PEO-CYTOP nanosheet.

b. Transmittance in the wavelength from 400 to 1000 nm of the light curable resin (NOA83H) used in the NIRE method.

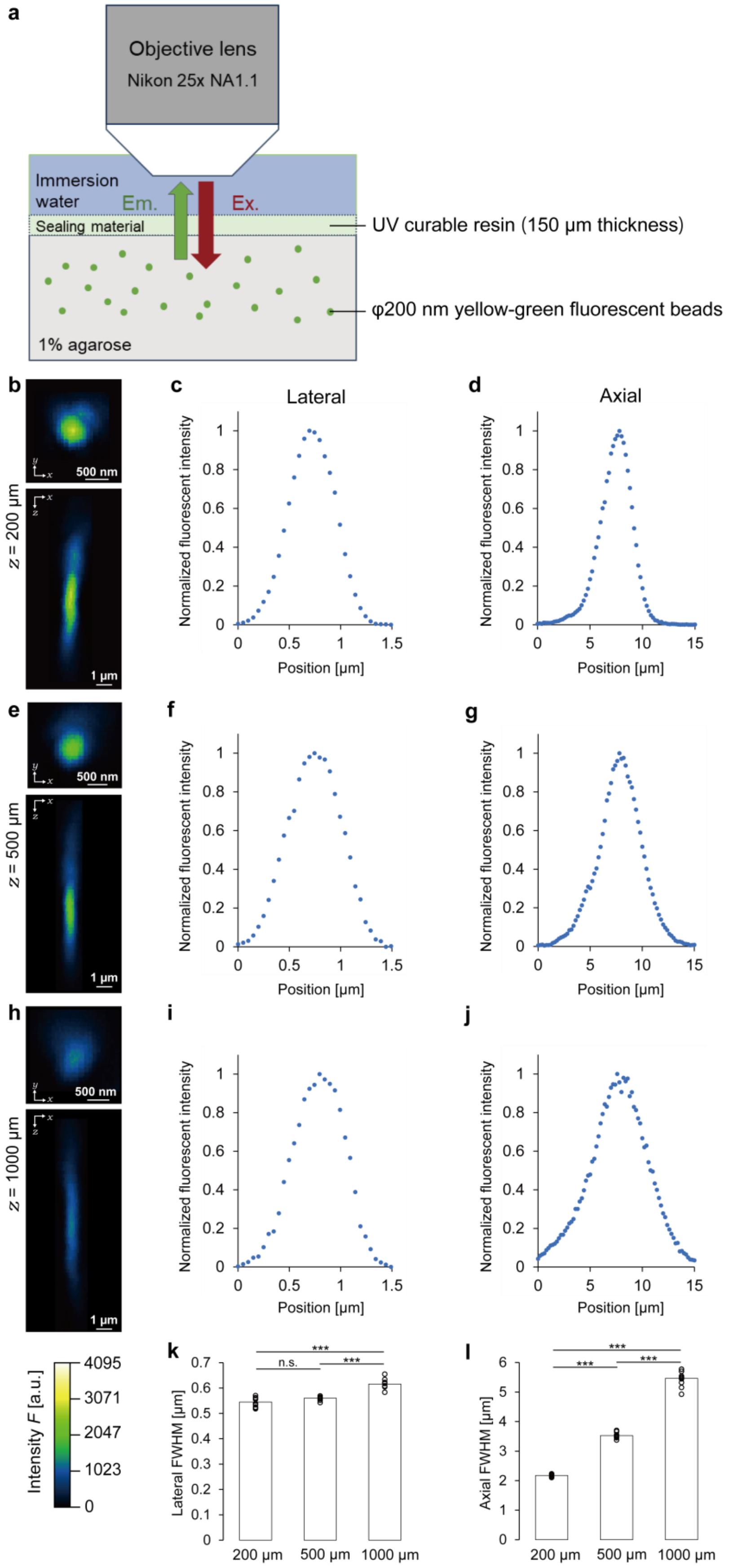
a
Objective lens
Nikon 25x NA1.1
Immersion water
Em.
Ex.
Sealing material
UV curable resin (150 μm thickness)
φ200 nm yellow-green fluorescent beads
1% agarose
b
c
Lateral
d
Axial
z = 200 μm
z = 500 μm
z = 1000 μm
500 nm
1 μm
e
f
g
h
i
j
Normalized fluorescent intensity
Position [μm]
Intensity F [a.u.]
4095
3071
2047
1023
0
k
l
n.s.
***
Lateral FWHM [μm]
Axial FWHM [μm]
200 μm
500 μm
1000 μm

75

Supplementary Figure 4. Evaluation of spatial resolution through the light curable resin using fluorescent beads in an agarose gel.

a. Schematic illustration of the measurement for evaluation of the spatial resolution through the light-curable resin.

b. Fluorescence images of 200-nm yellow-green beads at a depth of 200 µm acquired through the light-curable resin. Top image: *xy* plane. Bottom image: *xz* plane.

c. Lateral normalized fluorescence intensity of 200-nm yellow-green beads at a depth of 200 µm acquired through the light-curable resin.

d. Axial normalized fluorescence intensity of 200-nm yellow-green beads at a depth of 200 µm acquired through the light-curable resin.

e. Fluorescence images of 200-nm yellow-green beads at a depth of 500 µm acquired through the light-curable resin. Top image: *xy* plane. Bottom image: *xz* plane.

f. Lateral normalized fluorescence intensity of 200-nm yellow-green beads at a depth of 500 µm acquired through the light-curable resin.

g. Axial normalized fluorescence intensity of 200-nm yellow-green beads at a depth of 500 µm acquired through the light-curable resin.

h. Fluorescence images of 200-nm yellow-green beads at a depth of 1000 µm acquired through the light-curable resin. Top image: *xy* plane. Bottom image: *xz* plane.

i. Lateral normalized fluorescence intensity of 200-nm yellow-green beads at a depth of 1000 µm obtained through the light-curable resin.

j. Axial normalized fluorescence intensity of 200-nm yellow-green beads at a depth of 1000 µm obtained through the light-curable resin.

k. Average lateral FWHMs measured from the intensity profiles of bead images at depths of 200, 500, and 1000 µm; ***$p < 0.005$ by Welch’s t-test with Bonferroni correction. Error bars represent standard error of the mean.

l. Averaged axial FWHMs measured from the intensity profiles of bead images at depths of 200, 500, and 1000 µm; ***$p < 0.005$ by Welch’s t-test with Bonferroni correction. Error bars represent standard error of the mean.

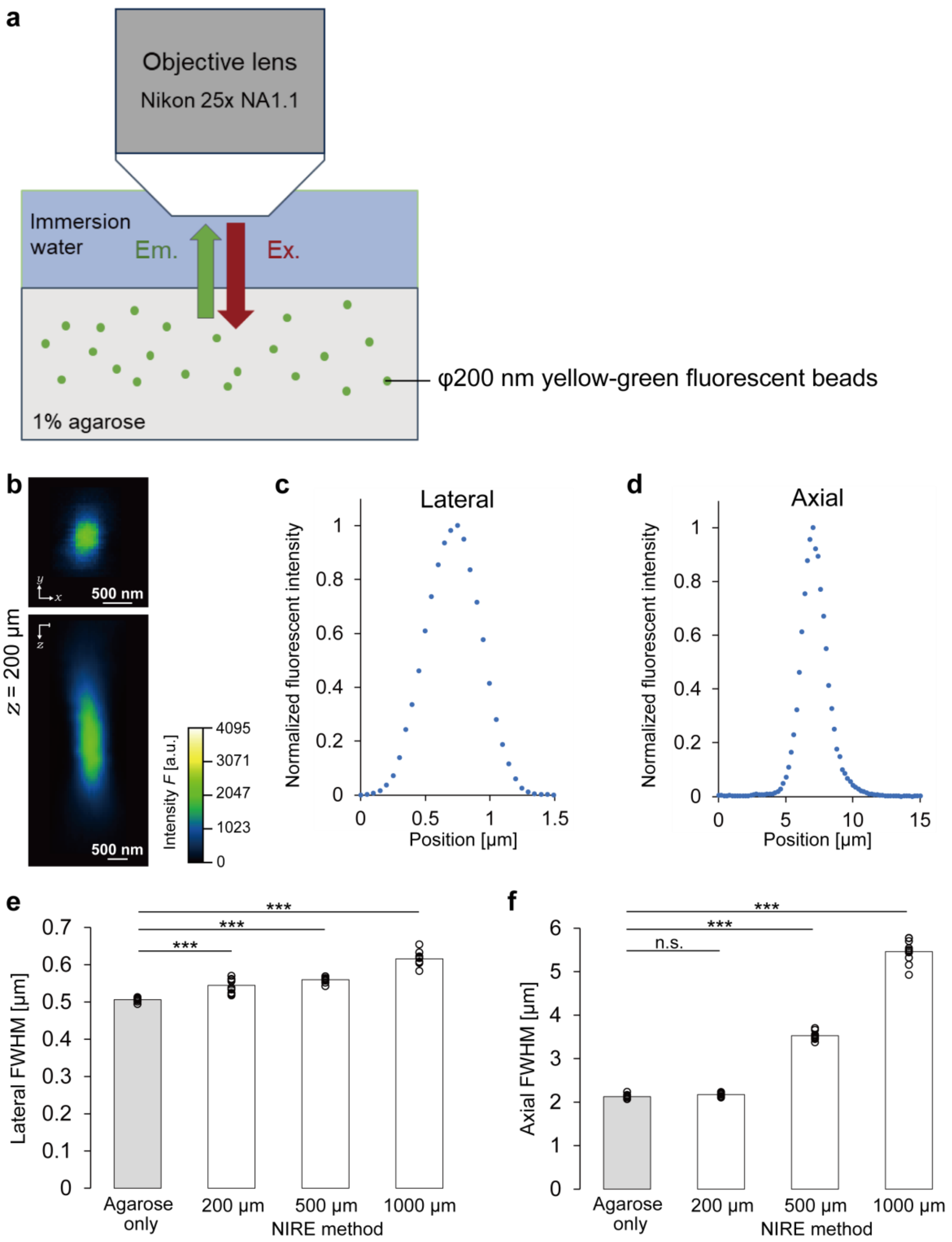

Supplementary Figure 5. Evaluation of spatial resolution using fluorescent beads in an agarose gel without the light curable resin.

a. Schematic illustration of the measurement for evaluation of the spatial resolution without any sealing materials.

b. Fluorescence images of 200-nm yellow-green beads at a depth of 200 µm acquired without any sealing materials. Top image: *xy* plane. Bottom image: *xz* plane.

c. Lateral normalized fluorescence intensity of 200-nm yellow-green beads at a depth of 200 µm acquired through a PEO-CYTOP nanosheet and light-curable resin.

d. Axial normalized fluorescence intensity of 200-nm yellow-green beads at a depth of 200 µm acquired through a PEO-CYTOP nanosheet and light-curable resin.

e. Average lateral FWHMs measured from the intensity profiles of bead images at depths of 200 µm without any sealing materials and 200, 500, and 1000 µm of Supplementary Fig.4; ***$p < 0.005$ by Welch's t-test with Bonferroni correction. Error bars represent standard error of the mean.

f. Average axial FWHMs measured from the intensity profiles of bead images at depths of 200 µm without any sealing materials and 200, 500, and 1000 µm of Supplementary Fig.4; ***$p < 0.005$ by Welch's t-test with Bonferroni correction. Error bars represent standard error of the mean.

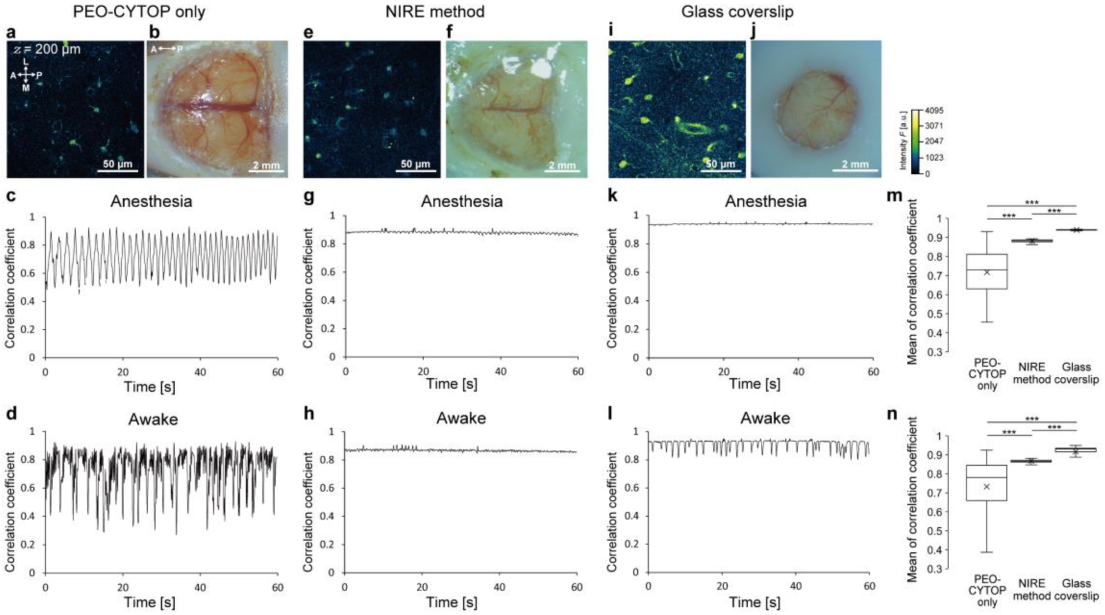

Supplementary Figure 6. Evaluation of motion artifacts in an awake mouse with cranial windows produced using the NIRE method.

a. Example two-photon image of SR101-labeled astrocytes acquired through a cranial window consisting of a PEO-CYTOP nanosheet. The directions are indicated as anterior (A), posterior (P), medial (M), and lateral (L).

b. The cranial window consisting of a PEO-CYTOP nanosheet on primary visual cortex (V1) of an adult wild-type mouse (same mouse as in **a**).

c. Time series of a correlation coefficient calculated from each frame in the anesthetized condition.

d. Time series of a correlation coefficient calculated from each frame in the awake condition.

e. Example two-photon image of SR101-labeled astrocytes obtained through a cranial window using the NIRE method (same mouse as in **a**).

f. A cranial window over V1 area produced using the NIRE method (same mouse as in **a**).

g. Time series of a correlation coefficient calculated from each frame in the anesthetized condition.

h. Time series of a correlation coefficient calculated from each frame in the awake condition.

i. Example two-photon image of SR101-labeled astrocytes obtained through a cranial window using the glass coverslip.

j. A cranial window using a glass coverslip with a diameter of 4.2 mm over V1 area (same mouse as in **i**).

k. Time series of a correlation coefficient calculated from each frame in the anesthetized condition.

l. Time series of a correlation coefficient calculated from each frame in the awake condition.

m. Mean of correlation coefficients for each sealing material under anesthesia; ***p < 0.005 by Welch's t-test with Bonferroni correction.

n. Mean of correlation coefficients for each sealing material in the awake state; ***p < 0.005 by Welch's t-test with Bonferroni correction.

Nikon Apo LWD 25×/1.10 NA water-immersion objective lens was used in (**a**), (**e**), (**i**).

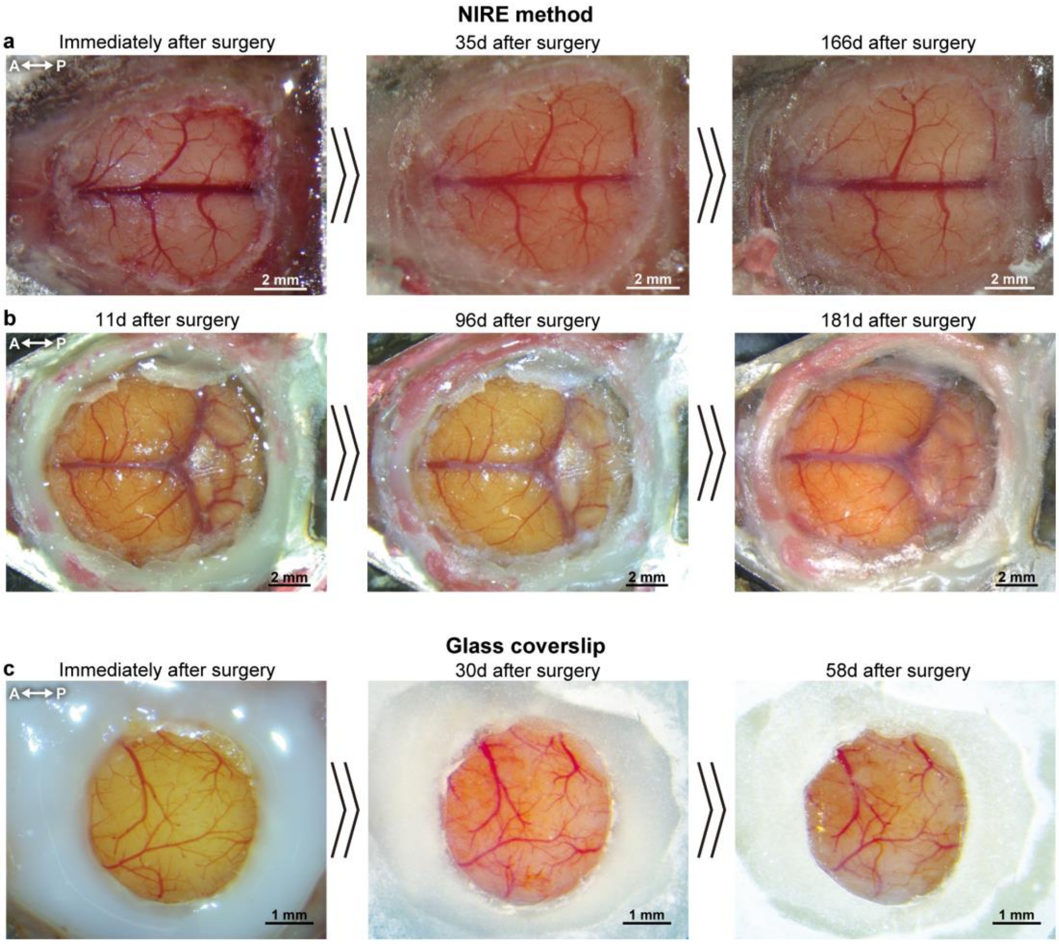

Supplementary Figure 7. Time-lapse photographs of the cranial window using the NIRE method or a glass coverslip.

a. Time-lapse photographs of the large cranial window in Figure 3-5 up to 166 days after surgery. The directions are indicated as anterior (A), and posterior (P).

b. Time-lapse photographs of the large cranial window in Figure 6 up to 181 days after surgery. The directions are indicated as anterior (A), and posterior (P).

c. Time-lapse photographs of the small cranial window using a glass coverslip with 4.2 mm diameter up to 58 days after surgery. The directions are indicated as anterior (A), and posterior (P).

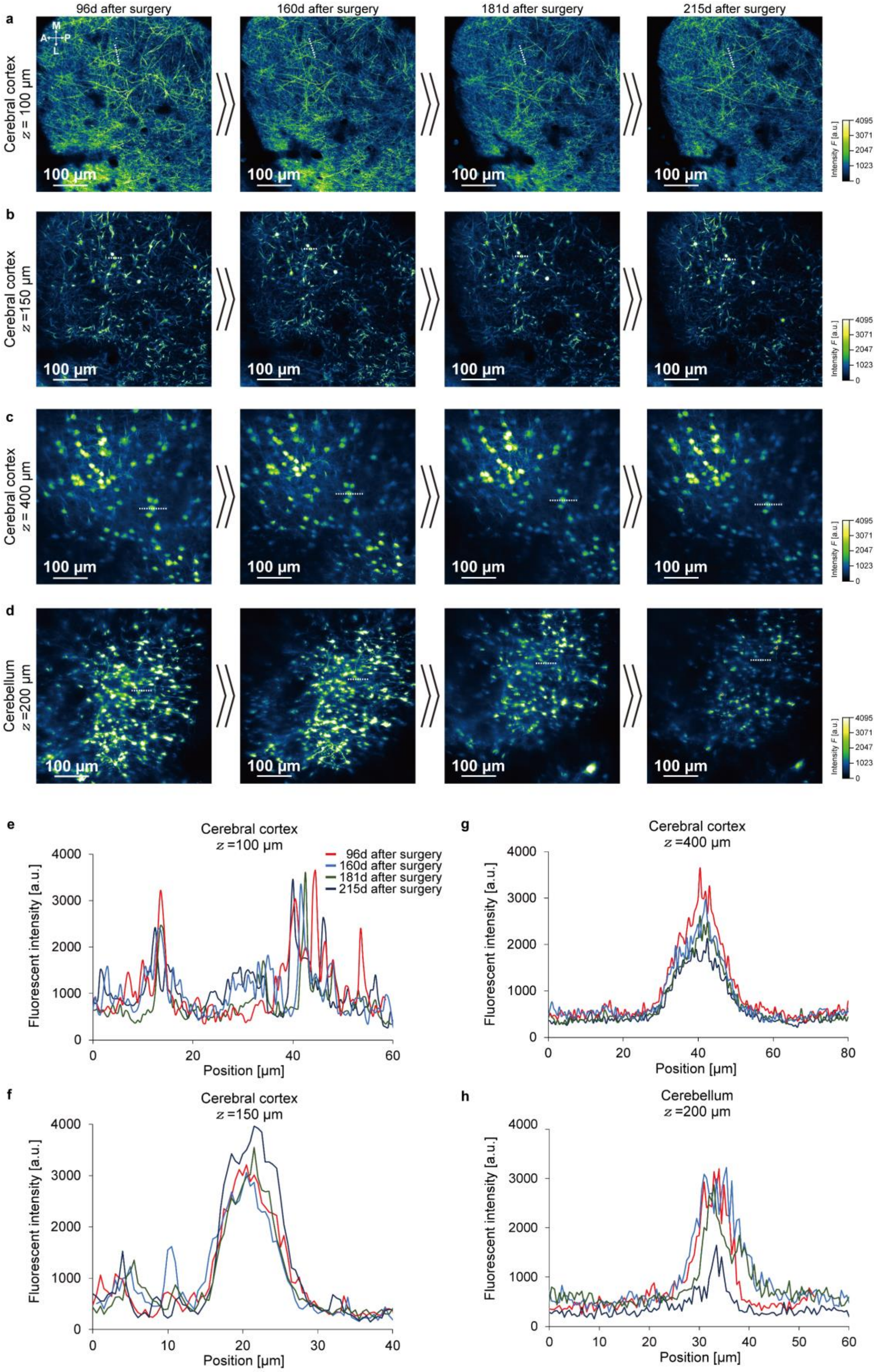
96d after surgery
160d after surgery
181d after surgery
215d after surgery
a
b
c
d
M
A
P
L
Cerebral cortex z = 100 μm
Cerebral cortex z =150 μm
Cerebral cortex z = 400 μm
Cerebellum z =200 μm
100 μm
Intensity F [a.u.]
4095
3071
2047
1023
0
e
Cerebral cortex z =100 μm
f
Cerebral cortex z =150 μm
g
Cerebral cortex z =400 μm
h
Cerebellum z =200 μm
Fluorescent intensity [a.u.]
Position [μm]

181

Supplementary Figure 8. Evaluation of fluorescent intensities in different depths through cranial windows produced using the NIRE method.

a. Time-lapse fluorescent images of the neurons in the cerebral cortex at the depth of 100 μm from the same mouse as in Fig. 6. The white dotted lines are indicated for the intensity profiles in (**e**). The directions are indicated as anterior (A), posterior (P), medial (M), and lateral (L).

b. Time-lapse fluorescent images of the neurons in the cerebral cortex at the depth of 150 μm from the same mouse as in Fig. 6. The white dotted lines are indicated for the intensity profiles in (**f**).

c. Time-lapse fluorescent images of the neurons in the cerebral cortex at the depth of 400 μm from the same mouse as in Fig. 6. The white dotted lines are indicated for the intensity profiles in (**g**).

d. Time-lapse fluorescent images of the neurons in the cerebellum at the depth of 200 μm from the same mouse as in Fig. 6. The white dotted lines are indicated for the intensity profiles in (**h**).

e. Fluorescence intensity profiles across the dendrites in the cerebral cortex indicated with lines in (**a**).

f. Fluorescence intensity profiles across the soma of the neuron in the cerebral cortex indicated with lines in (**b**).

g. Fluorescence intensity profiles across the soma of the neuron in the cerebral cortex indicated with lines in (**c**).

h. Fluorescence intensity profiles across the soma of the neuron in the cerebellum indicated with lines in (**d**).

Nikon Apo LWD 25×/1.10 NA water-immersion objective lens was used in (**a**)- (**d**).

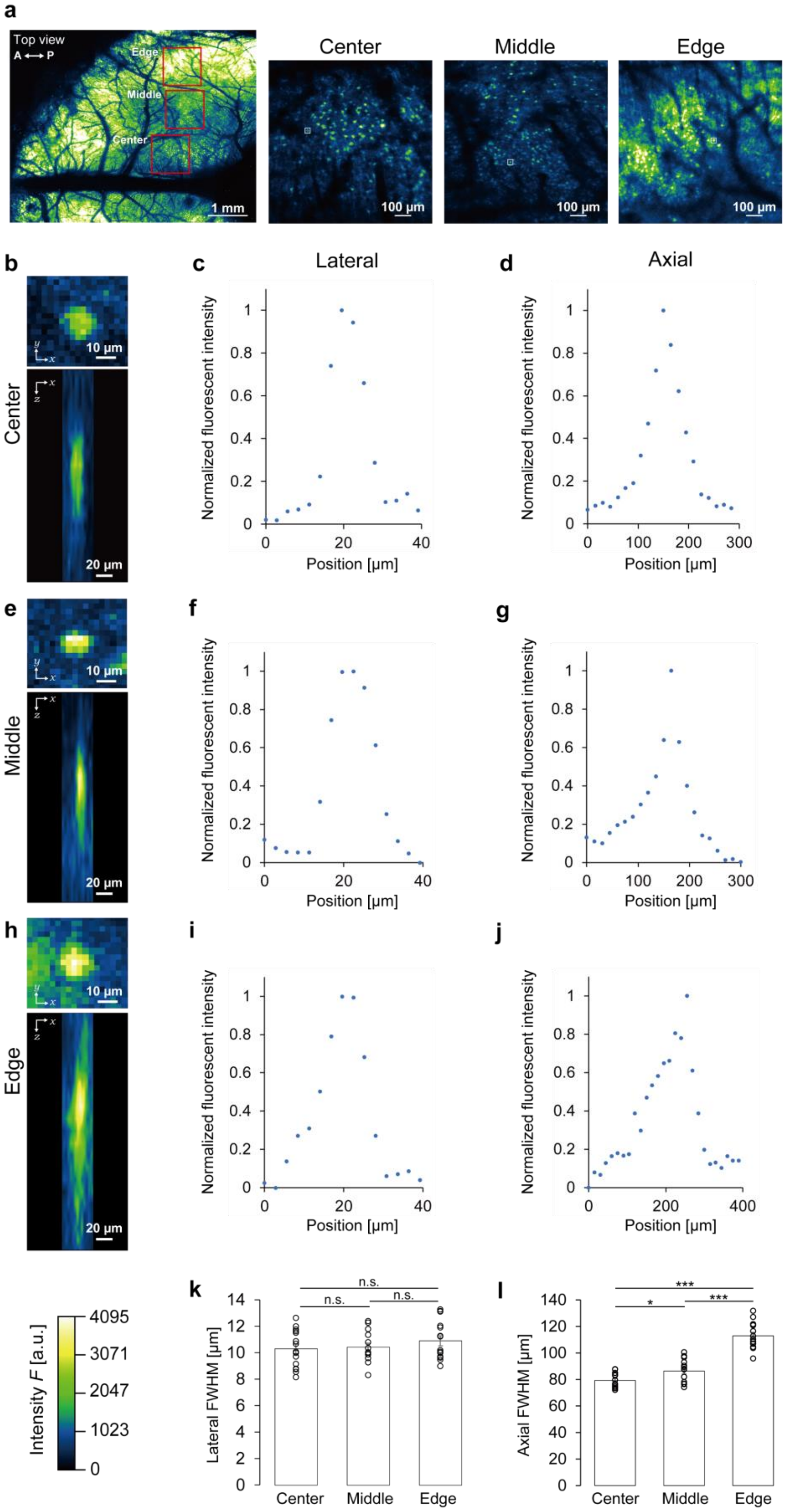
a
Top view
A ⟷ P
Edge
Middle
Center
1 mm
Center
Middle
Edge
100 μm
100 μm
100 μm
b
c
Lateral
d
Axial
Center
10 μm
20 μm
Normalized fluorescent intensity
Position [μm]
e
f
g
Middle
10 μm
20 μm
h
i
j
Edge
10 μm
20 μm
k
l
n.s.
n.s.
n.s.
*
***
***
Lateral FWHM [μm]
Axial FWHM [μm]
Center
Middle
Edge
Intensity F [a.u.]
4095
3071
2047
1023
0

Supplementary Figure 9. Evaluation of the spatial resolution of the neurons through the large cranial window from the cerebral cortex to the cerebellum.

a. Fluorescence image indicating each region of the target for the FWHM measurements using the same mouse as in Fig. 6. The directions are indicated as anterior (A) and posterior (P).

b. Fluorescence images of single neurons at the center of the large cranial window produced using the NIRE method indicated in (**a**). Top image: *xy* plane. Bottom image: *xz* plane.

c. Lateral normalized fluorescence intensity of single neurons at the center of the large cranial window produced using the NIRE method.

d. Axial normalized fluorescence intensity of single neurons at the center of the large cranial window produced using the NIRE method.

e. Fluorescence images of single neurons at the middle of the large cranial window produced using the NIRE method indicated in (**a**). Top image: *xy* plane. Bottom image: *xz* plane.

f. Lateral normalized fluorescence intensity of single neurons at the middle of the large cranial window produced using the NIRE method.

g. Axial normalized fluorescence intensity of single neurons at the middle of the large cranial window produced using the NIRE method.

h. Fluorescence images of single neurons at the edge of the large cranial window produced using the NIRE method indicated in (**a**). Top image: *xy* plane. Bottom image: *xz* plane.

i. Lateral normalized fluorescence intensity of single neurons at the edge of the large cranial window produced using the NIRE method.

j. Axial normalized fluorescence intensity of single neurons at the edge of the large cranial window produced using the NIRE method.

k. Average lateral FWHMs measured from the intensity profiles of single neurons at the center, middle, and edge of the cranial window; ***p < 0.005 by Welch's t-test with Bonferroni correction. Error bars represent standard error of the mean.

l. Average axial FWHMs measured from the intensity profiles of single neurons at the center, middle, and edge of the cranial window; *p < 0.05, ***p < 0.005 by Welch's t-test with Bonferroni correction. Error bars represent standard error of the mean.

Olympus XLFLUOR4X/340 4×/0.28 NA air-immersion objective lens was used in (**a**).

Fig. / Supplementary Fig.	Laser power (mW)	Imaging depth (μm)	Frame per seconds	Excitation wavelength (nm)	Objective lens	Fluorescent probe
Fig. 2b	15	0-600	0.5	960	Olympus XLFLUOR4X/340 4× /0.28 NA	EYFP
Fig. 2c-f	9-66	100-800	0.5	960	Nikon CFI75 LWD 25×/0.80 NA	EYFP
Fig. 4	71	150	3.9	950	Olympus XLFLUOR4X/340 4× /0.28 NA	jGCaMP7f
Fig. 5	33	150	7.7	950	Nikon Apo LWD 25×/1.10 NA	jGCaMP7f
Fig. 6b, Supplementary Fig. 9	13-58	0-600	0.5	960	Olympus XLFLUOR4X/340 4× /0.28 NA	EYFP
Fig. 6c,d, Supplementary Fig. 8a-c	7-54	100-400	0.5	960	Nikon Apo LWD 25×/1.10 NA	EYFP
Fig. 6e,f, Supplementary Fig. 8d	70	200	0.5	960	Nikon Apo LWD 25×/1.10 NA	EYFP
Supplementary Fig. 6	20	200	7.7	960	Nikon Apo LWD 25×/1.10 NA	SR101

Supplementary Table 1: The parameters used in each figure to perform *in vivo* imaging.
