# Supplementary material for: Large-scale cranial window for in vivo mouse brain imaging utilizing fluoropolymer nanosheet and light-curable resin
Source: Commun Biol. 2024 Mar 4;7:232. doi: 10.1038/s42003-024-05865-8 (PMC10912766; doi:10.1038/s42003-024-05865-8)
Supplement: Supplementary file 2 — Description of Supplementary Materials [file 42003_2024_5865_MOESM2_ESM.docx]

**Description of Additional Supplementary Files**

**File name:** Supplementary Data 1

**Description:** Source data underlying the graphs and charts.

**File name:** Supplementary Movie 1

**Description:** In vivo largescale two-photon imaging of neuronal activities using the NIRE Method. Related to Fig. 4

**File name:** Supplementary Movie 2

**Description:** In vivo calcium two-photon imaging of somata, dendrites, and spines using the NIRE Method. Related to Fig. 5.

**File name:** Supplementary Movie 3

**Description:** In vivo largescale two-photon imaging of neuronal structures from the cerebral cortex to cerebellum using the NIRE Method. Related to Fig. 6.
