# Supplementary material for: Large-scale cranial window for in vivo mouse brain imaging utilizing fluoropolymer nanosheet and light-curable resin
Source: Commun Biol. 2024 Mar 4;7:232. doi: 10.1038/s42003-024-05865-8 (PMC10912766; doi:10.1038/s42003-024-05865-8)
Supplement: Supplementary file 7 — Reporting Summary [file 42003_2024_5865_MOESM7_ESM.pdf]

nature portfolio

Corresponding author(s): Tomomi Nemoto

Last updated by author(s): Nov 10, 2023

Reporting Summary

Nature Portfolio wishes to improve the reproducibility of the work that we publish. This form provides structure for consistency and transparency in reporting. For further information on Nature Portfolio policies, see our Editorial Policies and the Editorial Policy Checklist.

Statistics

For all statistical analyses, confirm that the following items are present in the figure legend, table legend, main text, or Methods section.

n/a	Confirmed	
☐	☒	The exact sample size (n) for each experimental group/condition, given as a discrete number and unit of measurement
☐	☒	A statement on whether measurements were taken from distinct samples or whether the same sample was measured repeatedly
☐	☒	The statistical test(s) used AND whether they are one- or two-sided<br>*Only common tests should be described solely by name; describe more complex techniques in the Methods section.*
☒	☐	A description of all covariates tested
☐	☒	A description of any assumptions or corrections, such as tests of normality and adjustment for multiple comparisons
☐	☒	A full description of the statistical parameters including central tendency (e.g. means) or other basic estimates (e.g. regression coefficient) AND variation (e.g. standard deviation) or associated estimates of uncertainty (e.g. confidence intervals)
☐	☒	For null hypothesis testing, the test statistic (e.g. F, t, r) with confidence intervals, effect sizes, degrees of freedom and P value noted<br>*Give P values as exact values whenever suitable.*
☒	☐	For Bayesian analysis, information on the choice of priors and Markov chain Monte Carlo settings
☒	☐	For hierarchical and complex designs, identification of the appropriate level for tests and full reporting of outcomes
☒	☐	Estimates of effect sizes (e.g. Cohen's d, Pearson's r), indicating how they were calculated

Our web collection on statistics for biologists contains articles on many of the points above.

Software and code

Policy information about availability of computer code

Data collection	All two-photon images were obtained using a two-photon laser microscopy customized for in vivo imaging (A1R-MP+, Nikon, Japan). Stained mouse brain slices were examined using a confocal microscope (Leica TCS SP8 STED 3X FALCON, Leica, USA).
Data analysis	All two-photon images were achieved using NIS element software (Nikon, Japan). FOV displacement was assessed by calculating a correlation coefficient in MATLAB (2019b, The MathWorks, Inc., USA). Motion artifact in the intracellular Ca2+ imaging data were processed using the ECC image alignment algorithm (Evangelidis, G. D. & Psarakis, E. Z. Parametric image alignment using enhanced correlation coefficient maximization. IEEE Trans Pattern Anal Mach Intell 30, 1858–1865 (2008).). Fluorescent signals induced by changes in intracellular Ca2+ (ΔF/F) were segmented and extracted from time-lapse images using EZcalcium (Cantu, D. A. et al. EZcalcium: Open-Source Toolbox for Analysis of Calcium Imaging Data. Front Neural Circuits 14, 1–9 (2020).) . Image stitching was done by Fiji (ImageJ) .

For manuscripts utilizing custom algorithms or software that are central to the research but not yet described in published literature, software must be made available to editors and reviewers. We strongly encourage code deposition in a community repository (e.g. GitHub). See the Nature Portfolio guidelines for submitting code & software for further information.

Data

Policy information about availability of data

All manuscripts must include a data availability statement. This statement should provide the following information, where applicable:

- Accession codes, unique identifiers, or web links for publicly available datasets
- A description of any restrictions on data availability
- For clinical datasets or third party data, please ensure that the statement adheres to our policy

The data and code supporting the current study have not been deposited in a public repository but are available from the corresponding author on reasonable requests.

Human research participants

Policy information about studies involving human research participants and Sex and Gender in Research.

Reporting on sex and gender	*Use the terms sex (biological attribute) and gender (shaped by social and cultural circumstances) carefully in order to avoid confusing both terms. Indicate if findings apply to only one sex or gender; describe whether sex and gender were considered in study design whether sex and/or gender was determined based on self-reporting or assigned and methods used. Provide in the source data disaggregated sex and gender data where this information has been collected, and consent has been obtained for sharing of individual-level data; provide overall numbers in this Reporting Summary. Please state if this information has not been collected. Report sex- and gender-based analyses where performed, justify reasons for lack of sex- and gender-based analysis.*
Population characteristics	*Describe the covariate-relevant population characteristics of the human research participants (e.g. age, genotypic information, past and current diagnosis and treatment categories). If you filled out the behavioural & social sciences study design questions and have nothing to add here, write "See above."*
Recruitment	*Describe how participants were recruited. Outline any potential self-selection bias or other biases that may be present and how these are likely to impact results.*
Ethics oversight	*Identify the organization(s) that approved the study protocol.*

Note that full information on the approval of the study protocol must also be provided in the manuscript.

Field-specific reporting

Please select the one below that is the best fit for your research. If you are not sure, read the appropriate sections before making your selection.

☒ Life sciences ☐ Behavioural & social sciences ☐ Ecological, evolutionary & environmental sciences

For a reference copy of the document with all sections, see nature.com/documents/nr-reporting-summary-flat.pdf

Life sciences study design

All studies must disclose on these points even when the disclosure is negative.

Sample size	We did not calculate the sample size because we conducted the experiments to demonstrate the efficacy of novel method.
Data exclusions	No data was excluded in this analysis of this article.
Replication	For all in vivo experiments, we conducted the experiments using over 3 mice to confirm the reproducibility.
Randomization	We did not perform any randomization because we conducted the experiments to demonstrate the efficacy of novel method.
Blinding	Blinding was not possible for in vivo imaging in this study. In the analysis of immunostaining , we used identical parameters among the slices.

Reporting for specific materials, systems and methods

We require information from authors about some types of materials, experimental systems and methods used in many studies. Here, indicate whether each material, system or method listed is relevant to your study. If you are not sure if a list item applies to your research, read the appropriate section before selecting a response.

Materials & experimental systems

n/a	Involved in the study	
☐	☒	Antibodies
☒	☐	Eukaryotic cell lines
☒	☐	Palaeontology and archaeology
☐	☒	Animals and other organisms
☒	☐	Clinical data
☒	☐	Dual use research of concern

Methods

n/a	Involved in the study	
☒	☐	ChIP-seq
☒	☐	Flow cytometry
☒	☐	MRI-based neuroimaging

Antibodies

Antibodies used	Primary antibody anti-GFAP (FUJIFILM Wako Pure Chemical Corporation, Japan)<br>Secondary antibody Alexa Fluor 594 donkey anti-mouse IgG (Thermo Fisher Scientific, Waltham, MA, USA)
Validation	All commercial antibodies have been validated by the manufacturer for the species and assays used in this study. Validation data is available on the website of the manufacturers.<br><br>anti-GFAP https://labchem-wako.fujifilm.com/asia/product/detail/W01W0101-2728.html<br>Alexa Fluor 594 donkey anti-mouse IgG https://www.thermofisher.com/antibody/product/Donkey-anti-Mouse-IgG-H-L-Secondary-Antibody-Polyclonal/R37115

Animals and other research organisms

Policy information about studies involving animals; ARRIVE guidelines recommended for reporting animal research, and Sex and Gender in Research

Laboratory animals	Immunostaining and intracellular calcium imaging experiments were conducted on adult wild-type C57BL/6J mice (males and females over 8 weeks of age), while analyses of neuronal morphology were conducted on Thy1-EYFP-H (H-line) transgenic mice 59. All mice were housed under a 12 h/12 h light/dark cycle.
Wild animals	This study did not use wild animals.
Reporting on sex	Both female and male mice were used.
Field-collected samples	This study did not use samples collected from the field.
Ethics oversight	All of the animal studies were approved by the Institutional Animal Care and Use Committee of NIPS and Recombinant DNA Experiments Safety Committee of National Institute for Physiological Sciences.

Note that full information on the approval of the study protocol must also be provided in the manuscript.
